# Which Ion Dominates the Temperature and Pressure Response of Halide Perovskites and Elpasolites?

**DOI:** 10.1021/acs.jpclett.3c02403

**Published:** 2023-10-02

**Authors:** Loreta A. Muscarella, Huygen J. Jöbsis, Bettina Baumgartner, P. Tim Prins, D. Nicolette Maaskant, Andrei V. Petukhov, Dmitry Chernyshov, Charles J. McMonagle, Eline M. Hutter

**Affiliations:** † Inorganic Chemistry and Catalysis Group, Debye Institute for Nanomaterials Science and Institute for Sustainable and Circular Chemistry, Department of Chemistry, Utrecht University, Princetonlaan 8, 3584 CB Utrecht, The Netherlands; ‡ Physical and Colloid Chemistry, Debye Institute for Nanomaterials Science, Department of Chemistry, Utrecht University, Padualaan 8, 3584 CH Utrecht, The Netherlands; § Swiss−Norwegian Beamlines, European Synchrotron Radiation Facility, 71 Avenue des Martyrs, 38000 Grenoble, France

## Abstract

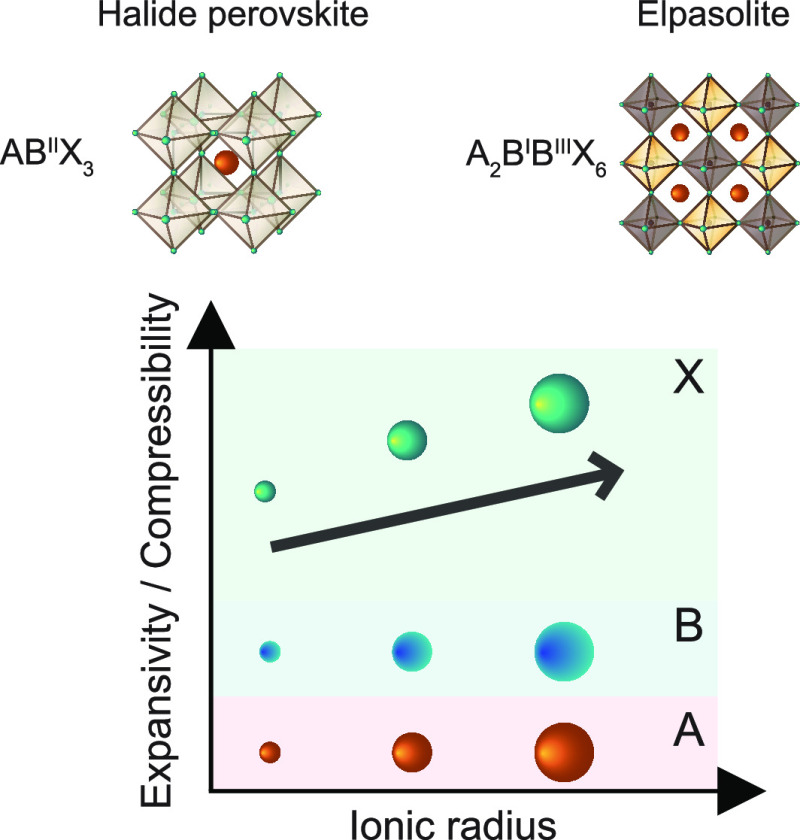

Halide perovskites and elpasolites are key for optoelectronic applications due to their exceptional performance and adaptability. However, understanding their crucial elastic properties for synthesis and device operation remains limited. We performed temperature- and pressure-dependent synchrotron-based powder X-ray diffraction at low pressures (ambient to 0.06 GPa) to investigate their elastic properties in their ambient-pressure crystal structure. We found common trends in bulk modulus and thermal expansivity, with an increased halide ionic radius (Cl to Br to I) resulting in greater softness, higher compressibility, and thermal expansivity in both materials. The A cation has a minor effect, and mixed-halide compositions show intermediate properties. Notably, thermal phase transitions in MAPbI_3_ and CsPbCl_3_ induced lattice softening and negative expansivity for specific crystal axes, even at temperatures far from the transition point. These results emphasize the significance of considering temperature-dependent elastic properties, which can significantly impact device stability and performance during manufacturing or temperature sweeps.

Lead halide perovskite and related lead-free elpasolite (i.e., double perovskite) semiconductors are widely investigated for a variety of optoelectronic applications, including photovoltaics,^1−4^ light-emitting diodes,^5,6^ photoredox catalysis,^7−10^ and radiation detection.^11−13^ The unprecedented interest in this class of materials can be understood from its excellent optoelectronic performance in combination with enormous chemical and structural flexibility. For instance, mixing halides in lead halide perovskites enables the absorption onset and emission wavelength over the entire visible range of the light spectrum to be tuned.^14^ In halide elpasolites, with the general formula A_2_B^I^B^III^X_6_, bandgap tunability can be obtained by mixing metals at the B^III^ site, such as Bi, Sb, In, or Fe,^15−17^ and to some extent by mixing halides (X site).^18−20^ In contrast to the plethora of studies of optoelectronic properties and applications of halide perovskites and elpasolites, there is still limited knowledge about their mechanical properties, such as compressibility and expansivity, and how these properties vary with the temperature and crystal axis. The solution-processed deposition of halide perovskites and elpasolites in the form of thin films onto substrates involves increased temperatures ranging from 100 to 200 °C. Additionally, the differences in the structural and thermal properties of substrates and halide perovskites, such as lattice parameters and thermal expansion coefficients, can lead to strain and deformations during thin film deposition. Previously, we and others have found that compression slows halide migration and that compression or strain can activate or suppress light-induced halide segregation.^21−24^ Thus, quantifying the extent to which this class of relatively soft materials responds to changes in temperature and pressure leading to stress-induced deformations is key for growing stable thin films of these perovskites and manipulating their optoelectronic properties. Previous studies that report on elastic properties used diamond anvil cells (DACs) to apply external pressure^23,25−31^ and hence probed high-pressure regimes (several to hundreds of gigapascals) and crystal phases that are far from relevant to their ambient crystal structure. The deformations induced in halide perovskite films during the solution-processed deposition are comparable to the exertion of mild pressure (<0.5 GPa).^32^ Therefore, hydraulic pressure techniques are needed to investigate how the crystal structure would respond in a more relevant pressure range. Given that the typical annealing temperatures applied during the deposition of halide perovskite thin films (100 °C) yield film stress levels of <0.06 GPa,^32^ our work, conducted through synchrotron-based powder X-ray diffraction (XRD), explored the structural properties of halide perovskites and elpasolites at increased pressures of ≤0.060 GPa, using a hydrostatic pressure cell. Importantly, this allowed us to determine the elastic properties of the materials in their crystal structures relevant to ambient conditions. The pressure-dependent measurements were performed between room temperature and 90 °C (298–363 K), a typical temperature range for thin film synthesis and photovoltaic operation conditions. We report the temperature-dependent bulk modulus for a wide variety of compositions, including MAPbCl_3_, -Br_3_, and -I_3_ (MA = methylammonium), mixed-halide variants thereof, CsPbCl_3_, -Br_3_, and -I_3_, and the elpasolites Cs_2_AgBiCl_6_ and -Br_6_ and mixed-halide and trivalent metal variants thereof. These materials were synthesized as powders using mechanochemical synthesis in a ball, which has been successfully used to make both perovskite and elpasolite materials.^10,33−36^ Powders are employed as a model system because they do not experience strain from the substrate. By using the same fabrication and measurement approach for a large number of compositions, we exclude effects from sample type or varying the synthesis procedure used in different laboratories, enabling us to draw robust and general conclusions about the fundamental properties and behaviors of these materials. Hence, we find some general trends in the elastic properties of halide perovskites and elpasolites. For all perovskite and elpasolite materials, we find that the iodide-based materials are substantially softer than their bromide- and chloride-based analogues and that the bulk modulus increases from I^–^ to Br^–^ to Cl^–^. The mixed-halide perovskites show bulk moduli between those of their pure compounds and, within the same crystal phase, exhibit a linear relation with the average halide radius. In addition, the bulk modulus appears to be constant in the investigated temperature range for all materials, provided that the materials remain in the same crystal structure. For both CsPbCl_3_ and MAPbI_3_, which undergo phase transitions at ∼325 and ∼330 K, respectively, we observe a decrease in the bulk modulus at temperatures close to the phase transition. In addition, as the elastic properties of noncubic perovskites are anisotropic, we also estimate the compressibility in different crystallographic directions. Finally, we find that some temperature-dependent phase transitions of halide perovskites are reflected in negative thermal expansion coefficients of certain crystal axes, already several tens of degrees below the transition temperature. The variation of thermal expansivity with a crystal axis (for noncubic perovskites) and crystal structure (i.e., temperature) could induce stress during synthesis or temperature cycling and, in turn affect the stability of the corresponding devices (e.g., ion migration). For instance, elpasolites are used in scintillation detectors, which convert high-energy radiation (such as γ rays) into visible light, allowing for their detection. By incorporating the knowledge of elastic properties, researchers can design elpasolite-based detectors that are more mechanically stable, ensuring a consistent performance over time in harsh environments. Additionally, the knowledge of the elastic properties can help identify potential phase transitions or structural instabilities in both lead halide perovskites and elpasolites, guiding researchers in avoiding conditions that might lead to undesirable changes in the crystal structure. Therefore, elastic properties should be considered for thin films in the design of optoelectronic devices of halide perovskites.

Microcrystalline powder samples of MAPb(Cl_1–*x*_Br_*x*_)_3_, MAPb(I_1–*x*_Br_*x*_)_3_, CsPbCl_3_, -Br_3_, and -I_3_, and several elpasolites (based on Cl^–^, Br^–^, Bi^3+^ and/or Fe^3+^, Sb^3+^, or In^3+^) were made via mechanochemical synthesis in a ball mill,^10,33−36^ as described in the Experimental Section in the Supporting Information. To quantify the effect of both pressure and temperature on the structural parameters of these halide perovskites and elpasolites, we performed pressure- and temperature-dependent powder XRD measurements using a synchrotron radiation source at European Synchrotron Radiation Facility (ESRF) beamline BM01.^37^ We used a hydrostatic pressure cell filled with a fluorinated inert liquid (FC-770) as a pressure-transmitting medium, and we performed pressure sweeps from 0.004 to 0.060 GPa at temperatures of 298, 318, 335, and 355 K. Additional details of the experimental setup can be found in the Experimental Section in the Supporting Information. This approach enables one to directly measure the variation of structural parameters as a function of pressure and temperature and thereby allows one to determine material properties such as the bulk modulus (*B*), compressibility (*K*), and thermal expansivity (α). Figure 1a shows selected reflections of the diffraction pattern of tetragonal (*I*4/*mcm* space group) MAPbI_3_ collected at 0.004 and 0.060 GPa and room temperature. The external compression shifts the diffraction peaks to larger 2θ values, while the crystal phase remains constant in the range of pressures explored. Figure 1b shows the diffraction pattern of MAPbI_3_ collected at 333 K. At this temperature, MAPbI_3_ is in a cubic crystal structure (*Pm*3̅*m* space group). Also in this case, we observe the shift of the reflections toward larger 2θ values without an indication of phase transition induced by external pressure. In panels c and d of Figure 1, we plot the lattice parameters of tetragonal MAPbI_3_ [room temperature, where *a* = *b* ≠ *c* (Figure 1c)] and cubic MAPbI_3_ [333 K, where *a* = *b* = *c* (Figure 1d)] as a function of external pressure. This pressure-induced variation of the lattice parameters was determined from refinements using the Rietveld method using FullProf Suite;^38^ i.e., the obtained diffractograms were fitted using pseudo-Voigt functions considering the unit cell dimensions and atomic coordinates as variables. Further details of the method are provided in the Experimental Section in the Supporting Information. In all cases, as expected, applying external pressure leads to a decrease in the lattice parameter.

**Figure 1:**
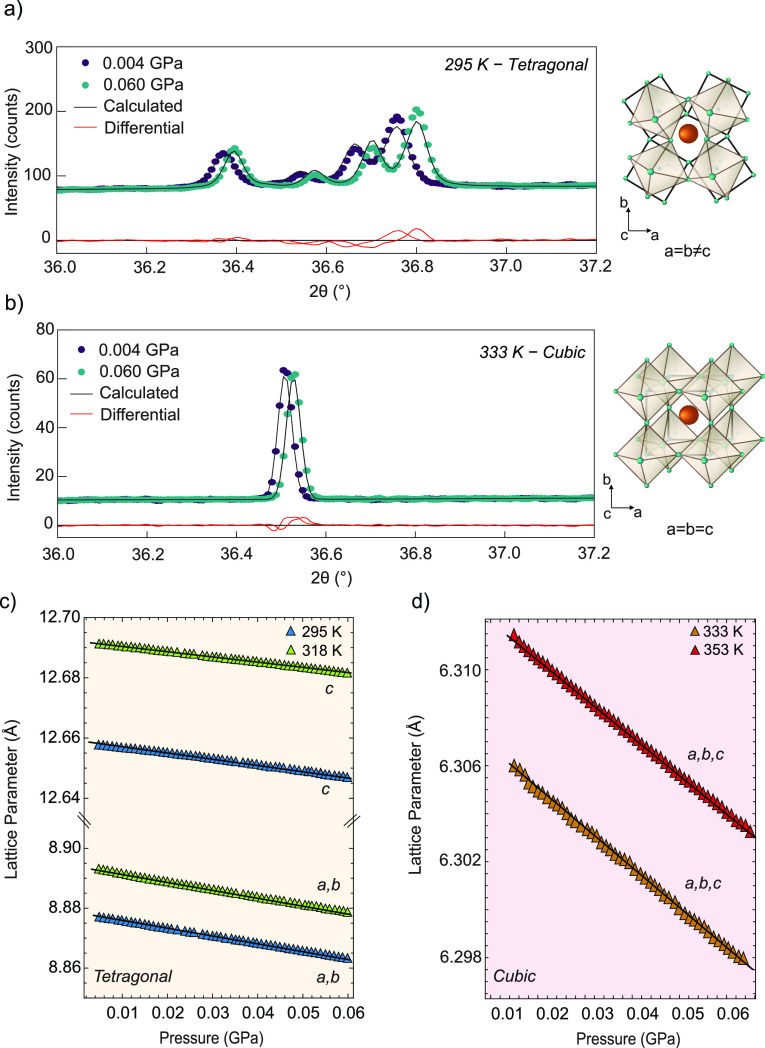
Selected reflections of the diffraction pattern of MAPbI_3_ collected at (a) room temperature and (b) 333 K, at 0.004 (purple) and 0.060 GPa (green), together with schematic drawings of the crystal structures. The calculated profile structure is colored black, and the residuals of the fit are colored red. Pressure-dependent lattice parameters of (c) tetragonal MAPbI_3_ (*I*4/*mcm* space group)^39^ collected at 295 and 318 K and (d) cubic MAPbI_3_ (*Pm*3̅*m* space group) collected at 335 and 355 K obtained from refinements using the Rietveld method. Fits are shown as black solid lines.

To investigate the role of the halide on the temperature-dependent elastic properties, we have performed a similar analysis of the mixed-halide perovskites MAPb(Cl_1–*x*_Br_*x*_)_3_ and MAPb(I_1–*x*_Br_*x*_)_3_, as reported in Figures S1–S7. Except for MAPbI_3_, which has tetragonal (*I*4/*mcm* space group) symmetry in the range of 298–330 K,^40,41^ these perovskites show cubic symmetry (*Pm*3̅*m* space group) under these conditions.^23,42^ For all single- and mixed-halide perovskites, we observed a linear decrease in volume with pressure for the temperatures explored. The isothermal equation of state for a solid is given by bulk modulus *B*:
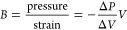
1where Δ*P*/Δ*V* is the derivative of pressure with respect to volume and *V* the volume at ambient pressure. By fitting this function to the pressure–volume trends (Figures S1 and S7), we estimated the bulk moduli (*B*) of the single halide (iodide, bromide, and chloride) and mixed-halide series MAPb(Cl_1–*x*_Br_*x*_)_3_ and MAPb(I_1–*x*_Br_*x*_)_3_. The results are shown in Figure 2a and listed in Table S1. In the entire temperature range of 298–355 K, MAPbBr_3_ (halide fraction *x* = 1) has a bulk modulus of 17 GPa, which gradually increases upon replacement of the Br^–^ with Cl^–^. An increase in the bulk modulus is associated with an increase in the stiffness of the material. At room temperature, MAPbCl_3_ has a bulk modulus of 20 GPa, which seems to be slightly lower (19 GPa) at increased temperatures. However, over the entire temperature range, the bromide-based perovskites are significantly softer than the perovskites containing chloride. The introduction of iodide leads to further softening of the perovskites, gradually decreasing the bulk modulus to 14 GPa. This softening of the bulk modulus with larger halides is consistent with previous single-crystal studies reporting a smaller Young’s modulus of the {100} crystal facet.^43^

**Figure 2:**
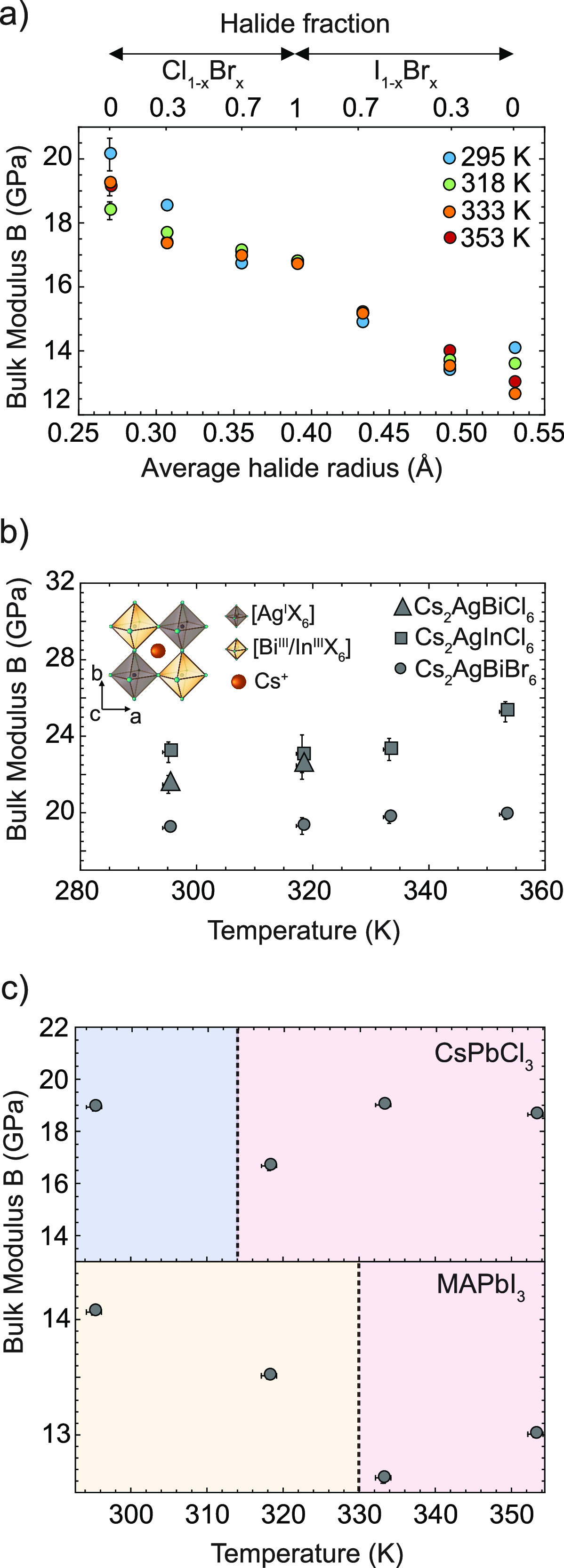
(a) Bulk moduli (*B*) of methylammonium (MA) lead mixed-halide perovskites as a function of the average halide radius and the mixing halide (Cl, left; Br, middle; I, right) ratio at 295, 318, 335, and 355 K. This shows that the Cl-based materials soften upon addition of Br^–^ and that I^–^ leads to further softening of the lattice. Note that except for MAPbI_3_, all of these perovskites exhibit a cubic symmetry, and their bulk modulus is roughly constant with temperature. (B) Bulk moduli of halide double perovskites Cs_2_AgBiBr_6_ (circles), Cs_2_AgInBr_6_ (squares), and Cs_2_AgBiCl_6_ (triangles) as a function of temperature. Also here, the Cl-based compounds are stiffer than Cs_2_AgBiBr_6_, with only minor temperature effects. All of the compositions are in the cubic phase, and no phase transition is observed. (C) Bulk moduli of MAPbI_3_ and CsPbCl_3_ as a function of temperature. The blue, yellow, and red regions correspond to the orthorhombic, tetragonal, and cubic phases, respectively. The dashed lines correspond to the temperature at which the phase transition occurs.

For the cubic systems, we observe a linear relation between the bulk modulus and the average halide radius as common for metal alloys (Figure S8).^44^ Such findings confirm previous assumptions with similar compositions^21^ but are in contrast with theoretical predictions for CsPb(I_1–*x*_Br_*x*_)_3_.^45^ To investigate the role of the MA^+^ cation in the halide perovskite and the role of the trivalent metal in the elpasolite structure with respect to their elastic properties, we determined the temperature-dependent bulk moduli of CsPbBr_3_ and -Cl_3_, as well as bulk moduli of Cs_2_AgBiBr_6_ and -Cl_6_ and Cs_2_AgInCl_6_. The results are shown in panels b and c of Figure 2 and Figures S9–S13, and numeric values are listed in Table S1. Even though the Cs-based perovskites have crystal structures different from the MA-based ones, the room-temperature bulk moduli of CsPbBr_3_ [*B* = 17.5 GPa (see Figure S12)] and CsPbCl_3_ [*B* = 19 GPa (see Figure 2c and Figure S13)] are almost identical to those of their MA-based counterparts (i.e., <1 GPa variation). These observations suggest that the mechanical properties are mostly defined by the framework of corner-sharing PbI_3_ (Cl_3_ or Br_3_) octahedra. Upon examination of Pb–X bonding from a perspective of electronegativity (EN), the EN values of X gradually decrease from chlorine to iodine (i.e., 3.16 for Cl > 2.96 for Br > 2.66 for I according to the Pauling scale). With a constant EN for Pb (1.87), the nature of bonding shifts from being primarily ionic for Cl to more covalent for I.^46^ Ionic compounds tend to possess hardness and brittleness, while covalent compounds lean toward softness and flexibility, which is reflected in the values of bulk moduli and compressibility we found in this study. A similar trend is observed upon comparison of the bromide- to chloride-based elpasolites. Also, the stiffer nature of the In–Cl elpasolites compared to Bi–Cl elpasolites could be due to its more ionic behavior arising from larger EN difference with the chloride (i.e., 1.78 for In vs 2.02 for Bi). Comparing the perovskites to the elpasolites, we observe that the replacement of Pb^2+^ with both Ag^+^ and a trivalent cation (Bi^3+^ or In^3+^) leads to a stiffening of the lattice, associated with larger bulk moduli, i.e., 22–23 GPa for those with Cl^–^ and 19 GPa for Cs_2_AgBiBr_6_,^27^ even though the Ag–X and Bi–X bonds are expected to be less ionic than the Pb–X bonds on the basis of their EN. Comprehending the full trend that encompasses alterations of metal, halide, and crystal structure therefore necessitates further investigation. However, overall, the effect of changing the cations is relatively small compared to the effect of changing the halides, so that the halide framework dominates the mechanical properties of halide perovskites and elpasolites rather than the A or B site cations. Interestingly, the elpasolite compositions exhibit bulk moduli on par with those of metal–organic framework (MOF) perovskites, such as [(CH_3_)_2_NH_2_][M(HCOO)_3_], where M = Mn^2+^, Fe^2+^, or Cu^2+^ (with bulk moduli ranging from 24 to 27 GPa^47^). Hence, the halide perovskites and elpasolites investigated in this study demonstrate bulk moduli significantly lower than those of other materials with similar structures, such as KCuF_3_ (*B* = 57 GPa^48^), LaMnO_3_ (*B* = 108 GPa^49^), and La_2_NiMnO_6_ (*B* = 179 GPa^50^). This renders them notably more susceptible to strain and external pressure in contrast to other materials.

Another general finding obtained from our study is that most of the bulk moduli only slightly change with temperature in the range from 298 to 355 K, in contrast with the expected decrease in *B* with temperature observed in oxide perovskites, and the expected increase calculated for tin-based halide perovskites such as CsSnI_3_.^51^ As one can see in panels a and b of Figure 2, the variation of the bulk modulus with the halide is larger than any temperature effect in this range. Two notable exceptions include CsPbCl_3_ and MAPbI_3_, which undergo temperature-dependent phase transitions between 298 and 330 K, shown in Figure 2c. Our data hint toward a softening of the materials (i.e., lower bulk modulus) close to their transition temperature (dotted lines). This finding is particularly relevant because temperature-dependent elastic properties may introduce stress during fabrication of halide perovskite and elpasolite thin films or during temperature cycling processes in operating devices.

Considering that the bulk modulus is a volume property, while the elastic properties of noncubic perovskites, such as tetragonal MAPbI_3_, are in general anisotropic and therefore depend on crystallographic direction, we derived the compressibility of the specific crystal axes by monitoring their compression as a function of applied pressure (e.g., *K*_*a*_, for compressibility along the *a*-axis):
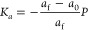
2where *a* is the lattice parameter and *a*_f_ – *a*_0_ the change in lattice parameter between the lattice parameter at the final (*a*_f_) and initial (*a*_0_) pressures. Hence, we determined the compressibility for the *a-*, *b-*, and *c-*axes for noncubic perovskites. The volumetric compressibility, *K*_V_, i.e., the susceptibility of a material to compress upon external applied pressure, is inversely proportional to *B* and is calculated as reported in Supporting Note 2, and the values are listed in Table S5. The compressibilities along the *a*-, *b*-, and *c*-axes for the compositions studied are reported in Tables S2–S4. Figure 3 shows that compression along the *a-* and *b-*axes of tetragonal MAPbI_3_ and orthorhombic CsPbCl_3_ is significantly larger than that for the *c*-axis. In addition, we observe that the compressibility of *c* increases significantly in CsPbCl_3_ during the orthorhombic-to-cubic phase transition as well as in MAPbI_3_ during the tetragonal-to-cubic phase transition, as it becomes identical to those of the *a-* and *b-*axes. In orthorhombic CsPbBr_3_, we find that compression along the *a-* and *c*-axes is significantly larger than that for the *b*-axis. Additional pressure studies using an extended temperature range are needed to determine the origin of the observed axis-dependent compressibility. For CsPbBr_3_, we observe a gradual increase in the compressibility of the *b*- and *c*-axes with temperature, in combination with swapping of the compressibility of the *a-* and *b-*axes at 353 K. This phenomenon may occur in the case of an anti-isostructural phase transition,^52,53^ although more measurements are needed to conclusively show whether such a transition exists in CsPbBr_3_. To investigate the relation between the pressure response and thermal expansivity, we further measured the temperature-dependent XRD of single-halide perovskites (MAPbI_3_, MAPbBr_3_, MAPbCl_3_, CsPbBr_3_, and CsPbCl_3_) and elpasolites (Cs_2_AgBiBr_6_, Cs_2_AgBiCl_6_, and Cs_2_AgInCl_6_) between 100 and 400 K. A similar Rietveld refinement was used to study the temperature dependence of *a*-, *b*-, and *c*-axes. The results are shown in Figure 4, whereas the lattice parameters reported in pseudocubic notation are reported in Figure S14. Here, blue areas represent orthorhombic (*Pnmb* space group), yellow areas tetragonal (*I*4/*mcm* space group), and red areas cubic symmetry (*Pm*3̅*m* space group). Although similar experiments have been reported for a selection of these compositions,^40,42,54,55^ we here compare the thermal expansivities of all compositions using identical synthesis and measurement conditions and link the thermal response to the elastic properties of the same materials.

**Figure 3:**
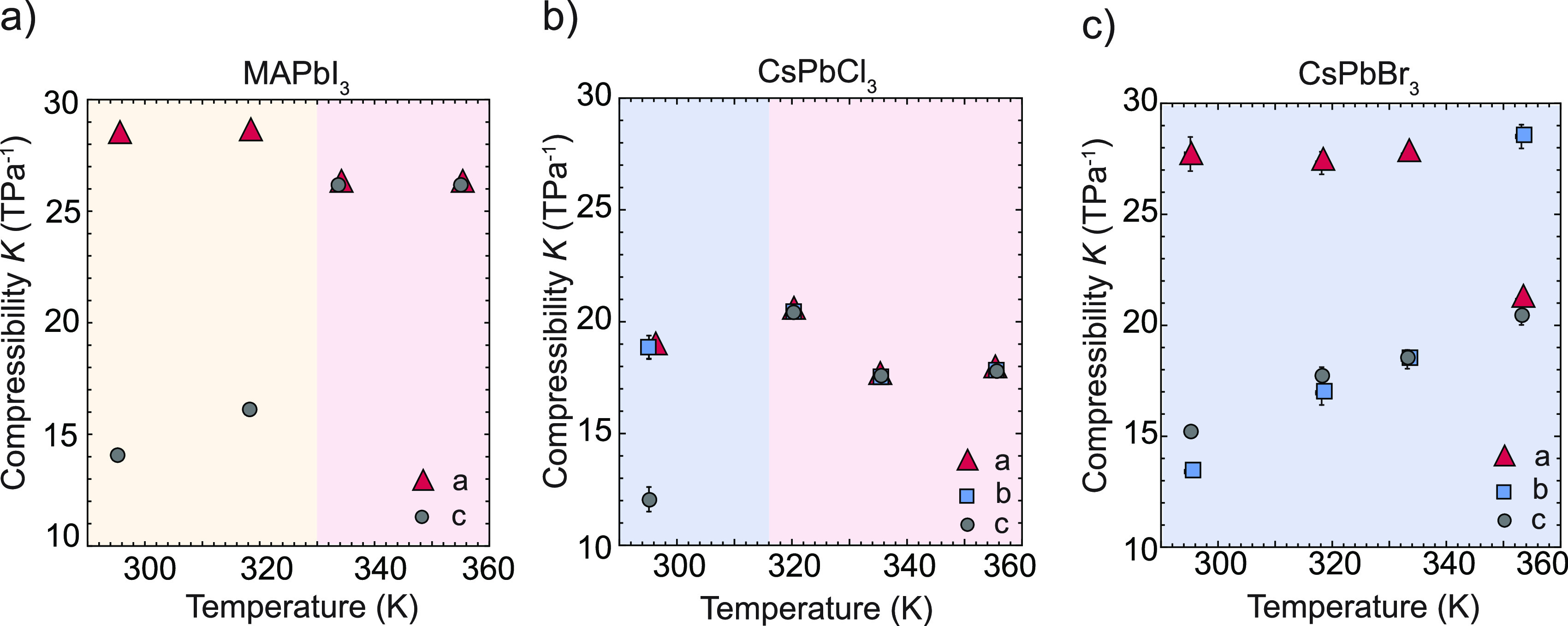
(a) Compressibility *K* of the crystal axes in the tetragonal (yellow) and cubic (red) phases of MAPbI_3_. The compressibility of the *c*-axis is smaller than that of the *a*-axis in the tetragonal phase. (b) Compressibility *K* of the crystal axes in the orthorhombic (blue) and cubic (red) phases of CsPbCl_3_. The compressibility of the *c*-axis is smaller than those of the *a*- and *b*-axes in the orthorhombic phase. (c) Compressibility *K* of the crystal axes in the orthorhombic (blue) phase. The compressibility of the *b*-axis is smaller than those of the *a-* and *c-*axes in the tetragonal phase up to 353 K where an abrupt change is observed. At 353 K, the compressibility of the *b-*axis is larger than those of the *a*- and *c-*axes. Error bars are, in some cases, smaller than the data point size.

**Figure 4:**
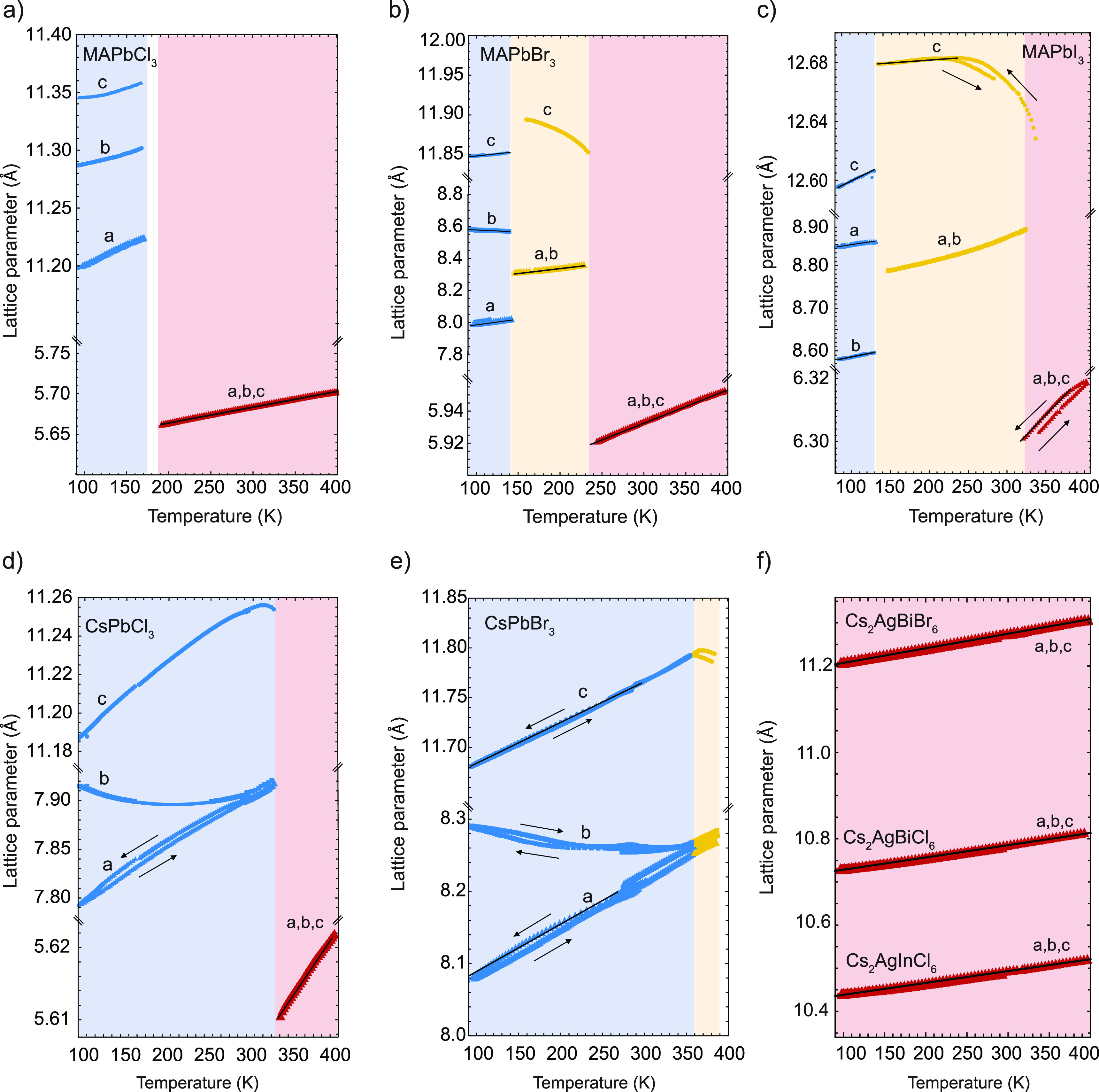
Temperature-dependent lattice parameters of (a) MAPbCl_3_, (b) MAPbBr_3_, (c) MAPbI_3_, (d) CsPbCl_3_, (e) CsPbBr_3_, and (f) Cs_2_AgBiBr_6_, Cs_2_AgBiCl_6_, and Cs_2_AgInCl_6_ obtained from profile Rietveld refinement of synchrotron XRD patterns collected between 90 and 400 K. The blue, yellow, and red regions correspond to the orthorhombic, tetragonal, and cubic phases, respectively. The white regions correspond to the temperature range in which two phases coexist in the XRD patterns. The black arrows indicate the direction of temperature variation, i.e., cooling (arrow down) and heating (arrow up). Solid lines represent the fits for the linear thermal expansivity.

Comparing the different halide perovskites and elpasolites in cubic symmetry (red areas), we observe an increase in lattice parameters as a function of temperature, associated with a positive thermal expansivity, as shown in Table 1. In line with the halide-dependent trend in bulk modulus, for all compositions studied here the thermal expansivity is observed to increase in the following order due to the softness of the material: Cl < Br < I. We observe that the elpasolites are less expandable with temperature than the lead halide perovskites, with thermal expansivities of 2.86 × 10^–5^ K^–1^ for Cs_2_AgBiBr_6_ and 2.42 × 10^–5^ K^–1^ for Cs_2_AgBiCl_6_. This observation is consistent with their larger bulk moduli, showing that the elpasolite structure is more resistant to changes in both temperature and pressure. Furthermore, the absolute values are close to recent theoretical predictions.^56^ Note that previous studies of Cs_2_AgBiBr_6_ thin films have reported a cubic-to-tetragonal phase transition at 122 K.^57^ The absence of this phase transition (at least down to 100 K) for the mechanochemically synthesized powders studied here may be related to differences in strain between the powders and thin films. Note that Cs_2_AgBiBr_6_ stands out as the sole exception; the other compositions exhibit the expected phase transitions at their respective temperatures.

**Table 1:** Thermal Expansivities along All Crystal Axes for All Compositions^a^

	orthorhombic (×10^–5^ K^–1^)		tetragonal (×10^–5^ K^–1^)		cubic (×10^–5^ K^–1^)
MAPbI_3_	*a*	3.81	*a* = *b*	function^b^	4.04
	*b*	2.02			
	*c*	5.81	*c*	0.34	
MAPbBr_3_	*a*	9.04	*a* = *b*	6.99	3.53
	*b*	–2.79			
	*c*	1.00	*c*	function^b^	
MAPbCl_3_	*a*	function^b^	–		3.51
	*b*	function^b^			
	*c*	function^b^			
CsPbBr_3_	*a*	7.68	–		3.27^c^
	*b*	function^b^			
	*c*	3.51			
CsPbCl_3_	*a*	function^b^	**–**		3.02
	*b*	function^b^			
	*c*	function^b^			
Cs_2_AgBi(Br_0.33_I_0.67_)_6_	–		–		5.08^c^
Cs_2_AgBiBr_6_	–		–		2.86
Cs_2_AgBiCl_6_	–		–		2.42
Cs_2_AgInCl_6_	–		–		2.49
Cs_2_Ag(Bi_0.5_In_0.5_)Br_6_	–		–		2.84^c^
Cs_2_Ag(Bi_0.5_Sb_0.5_)Br_6_	–		–		3.34^c^
Cs_2_Ag(Bi_0.9_Fe_0.1_)Br_6_	–		–		3.16^c^

a If the temperature response is nonlinear, the experimental data are fitted using a second-order polynomial and denoted by “function”. The optimized parameters of these fits are given in Supporting Note 3 and Figures S15–S19. As the temperature range in which the materials are orthorhombic, tetragonal, or cubic varies with composition, the reported (functions of) thermal expansivity is valid only in that specific temperature range.

b Nonlinear expansivity (see Supporting Note 3).

c Data collected with a lab-based X-ray diffractometer.

Additional lab-based temperature-dependent XRD on elpasolite with mixed trivalent metals, i.e., Cs_2_Ag(Bi_0.5_Sb_0.5_)Br_6_, Cs_2_Ag(Bi_0.5_In_0.5_)Br_6_, and Cs_2_Ag(Bi_0.9_Fe_0.1_)Br_6_, and mixed halides such as Cs_2_AgBi(Br_0.33_I_0.67_)_6_ (Supporting Note 4 and Figures S20 and S21) indicates higher thermal expansivity for elpasolites containing I^–^ ions, identical to the lead-based perovskites (see Table 1). Previous work already showed a minor role of the Cs cation on the thermal expansivity in halide elpasolites,^58^ and we here also find that the metal cation does not affect the thermal expansivity. For phases with symmetry lower than cubic, i.e., orthorhombic (blue) and tetragonal (yellow), thermal expansivity is anisotropic. Interestingly, the axis-dependent thermal expansivity and compressibility in the tetragonal crystal structure follow the same trend (i.e., the *c*-axis is less expandable and more compressible than the *a*- and *b*-axes). On the contrary, in the orthorhombic crystal phase we observe no such relation, suggesting that thermal expansivity and compressibility are decoupled in this phase. An additional consideration is that some lattice parameters have nonlinear temperature-dependent thermal expansivity. For example, the axes of orthorhombic CsPbBr_3_ and CsPbCl_3_ and tetragonal MAPbI_3_ show a nonlinear dependence of lattice parameter with temperature, so that the thermal expansivity becomes temperature-dependent (see Supporting Note 3 and Table S6). Notably, in all halide perovskites studied here, the *c-*axis of the tetragonal phase has a negative thermal expansivity in at least part of the temperature range. According to Landau theory, such a behavior reflects bi-linear coupling of the order parameter and spontaneous lattice strain.^59^ Considering that negative thermal expansivity values (i.e., thermal contraction) are mainly observed at temperatures “close” to thermal phase transitions, it seems likely that these two phenomena are related. Previous work has reported similar behavior for MAPbI_3_ and formamidinium lead iodide (FAPbI_3_) perovskites^60−62^ as well as several oxide-based materials that undergo phase transitions.^63^ For CsPbBr_3_ and CsPbCl_3_, the temperature range with negative thermal expansivity for the *c*-axis is relatively small (20–40 K). In contrast, for MAPbBr_3_ and MAPbI_3_, the negative thermal expansivity of the *c*-axis is already observed 100 K below the tetragonal–cubic phase transition. This observation, together with temperature-dependent bulk modulus from Figure 2a, shows that the range in which the phase transition is affecting thermal and elastic properties is substantially broader than previously thought on the basis of the (much smaller) temperature range in which crystal phases coexist.^40^ This has major implications for synthesizing thin films on substrates with thermal expansivities different from those exhibited by the perovskite, where the temperature gradient during cooling from annealing to room temperature will introduce strain into the material.

We determined temperature-dependent elastic properties of several halide perovskites (namely, single and mixed halide) and elpasolite (namely, mixed halides and mixed trivalent metals) from pressure- and temperature-dependent diffraction experiments using a synchrotron radiation source. We used temperatures ranging from 298 to 363 K (i.e., ambient to 90 °C), which is a typical temperature range for thin film synthesis and photovoltaic operation conditions. To obtain information relevant to ambient phases, we explored pressures from ambient to 0.06 GPa. We find that the elastic properties and the thermal expansivity in both halide perovskites and elpasolites are dominated by the halide framework, with lower bulk moduli for larger halide radius size (Cl < Br < I). It is worth noting that in both lead halide perovskites and elpasolites, the elastic properties are intimately connected with the type of bond formed between the metal and the halide. As the halide undergoes a transition from the more electronegative Cl^–^ to the less electronegative I^–^, the bonding characteristics evolve from being predominantly ionic to becoming increasingly covalent. This shift influences the behaviors of the materials. Ionic compounds often exhibit traits of hardness and brittleness, whereas covalent compounds tend to demonstrate qualities of softness and flexibility. These inherent properties are effectively mirrored in the bulk moduli and compressibility values, as found in our research. On the contrary, the monovalent cation and the trivalent metal have a minor role in determining elastic properties. The bulk modulus of these materials remains constant within the investigated temperature range provided that the crystal structure remains unchanged. On the contrary, we observe that thermal phase transitions lead to a decrease in the bulk modulus (i.e., softening of the material), as exemplified by MAPbI_3_ and CsPbCl_3_. For noncubic systems, in which the elastic properties are anisotropic, we obtained axis-dependent compressibility. Our results suggest that the *c*-axis is much harder than the *a*- and *b*-axes in tetragonal MAPbI_3_ and orthorhombic CsPbCl_3_, whereas the *b*-axis is the hardest axis in CsPbBr_3_. Furthermore, we find that some temperature-dependent phase transitions of halide perovskites are accompanied by negative thermal expansivity of certain crystal axes, already several tens of degrees below the phase transition temperature. This observation shows that the range in which the phase transition is affecting thermal and elastic properties is substantially broader than previously thought on the basis of the (much smaller) temperature range in which phases coexist. Altogether, these findings have major implications for synthesizing thin films at substrates with thermal expansivities different from those of the perovskite and elpasolite, where the temperature gradient during the synthesis and the temperature cycling during device operation will introduce strain into the material, consequently exerting a noteworthy influence on the functionality of related devices. Hence, acquiring a deep comprehension of the temperature-dependent elastic properties in these materials is essential for bolstering the advancements in strain engineering that has proven to be a potent tool for manipulating both the optical properties and the stability of perovskite thin films.

## Data Availability

Data and fit procedures reported in this study can be accessed at https://data.esrf.fr/doi/10.15151/ESRF-ES-1022932247 (raw data) and https://data.esrf.fr/doi/10.24416/UU01-W60H58 (processed data) and are available under a CC-BY Creative Commons Attribution 4.0 International license.

## Supporting Information Available

Experimental details, materials, and methods; lattice parameters as a function of pressure, *P–V* curves, temperature-dependent bulk moduli, and axis-dependent compressibility for all of the compositions explored; *in situ* temperature-dependent XRD of several elpasolites; tables of temperature-dependent bulk moduli, axis-dependent compressibility, and temperature-dependent expansivity; lab-based temperature-dependent X-ray diffraction for additional elpasolites; calculation of volumetric and axis-dependent thermal expansivity and compressibility; and calculation of the bulk moduli (PDF)
Transparent Peer Review report available (PDF)
