# Supplementary material for: Which Ion Dominates the Temperature and Pressure Response of Halide Perovskites and Elpasolites?
Source: J Phys Chem Lett. 2023 Oct 2;14(40):9042–51. doi: 10.1021/acs.jpclett.3c02403 (PMC10577787; doi:10.1021/acs.jpclett.3c02403)
Supplement: Supplementary file 1 — jz3c02403_si_001.pdf [file jz3c02403_si_001.pdf]

Supporting Information to

Which Ion Dominates Temperature and Pressure Response of Halide Perovskites and Elpasolites?

Loreta A. Muscarella,[1#*] Huygen J. Jöbsis,[1#] Bettina Baumgartner,[1] P. Tim Prins,[1] D. Nicolette Maaskant,[1] Andrei V. Petukhov,[2] Dmitry Chernyshov,[3] Charles J. McMonagle,[3] and Eline M. Hutter[1*]

1. Inorganic Chemistry and Catalysis group, Debye Institute for Nanomaterials Science and Institute for Sustainable and Circular Chemistry, Department of Chemistry, Utrecht University, Princetonlaan 8, 3584 CB Utrecht, the Netherlands
2. Physical and Colloid Chemistry, Debye Institute for Nanomaterials Science, Department of Chemistry, Utrecht University, Padualaan 8, 3584 CH Utrecht, the Netherlands
3. Swiss–Norwegian Beamlines, European Synchrotron Radiation Facility, 71 Avenue des Martyrs, 38000 Grenoble, France

# These authors contributed equally

* Correspondence should be addressed to E.M.Hutter@uu.nl and loretaangela.muscarella@gmail.com

Experimental Section

Materials

The perovskite and elpasolite powders are prepared using lead (II) iodide (PbI_2; TCI, purity 99.99%, trace metals basis), methylamine hydroiodide (MAI; TCI, purity >99%), lead (II) bromide (TCI, purity 98%), and methylamine hydrobromide (MABr; purity TCI, >98%), lead (II) chloride ($PbCl_2$; TCI, purity >99%) and methylamine hydrochloride (MACl; TCI, purity >98%), cesium iodide (CsI; TCI, purity > 99%), cesium bromide (CsBr; TCI, purity > 99%), cesium chloride (CsCl; TCI, purity > 99%), silver (I) chloride (AgCl; Merck, purity 99.999% trace metals basis), silver (I) bromide (AgBr; Alfa Aesar, Premion®, purity 99.998% metals basis), bismuth (III) iodide (BiI_3, Merck, purity 99%), bismuth (III) bromide ($BiBr_3$; Merck, purity ≥ 98%), bismuth (III) chloride ($BiCl_3$; Merck, purity 99.998% trace metals basis), indium (III) bromide ($InBr_3$, Alfa Aesar, purity 99.99% (metal basis)), indium (III) chloride ($InCl_3$; TCI, purity >99.0%) and, antimony (III) bromide ($SbBr_3$, Alfa Aesar, purity ultra-dry 99.999% (metal basis)), iron (III) bromide ($FeBr_3$, Alfa Aesar, purity >98%). The precursor powders are obtained weighing the components with the desired molar stoichiometric ratio without further purification.

Perovskite powder preparation

Methylammonium-based perovskite powders are prepared using mechanochemical synthesis by grinding the precursor salts in a ball mill (Retsch Ball Mill MM-400) using a grinding jar of 10 ml and two stainless steel balls (⌀10 mm) for 60 minutes at 30 Hz. $CsPbX_3$ (X= I, Br, Cl) and elpasolite powders are prepared milling at 30 Hz for 90 minutes. No annealing is performed after the mechanochemical synthesis.

Pressure and temperature dependent powder X-ray diffraction

Pressure and temperature dependent powder X-ray diffraction data were collected at the Swiss–Norwegian Beamline BM01 (ESRF, Grenoble), with a $\lambda = 0.9590$ Å.[1] The powdered samples were loaded into a sapphire capillary pressure cell with a 1 mm external and 0.6 mm internal diameter (CRYTUR, spol. S r.o., Czech Republic).[2] The pressure cell was filled with a fluorinated inert liquid (FC-770, 3M) as a pressure-transmitting medium and the pressure was swept from 0.004 to 0.060 GPa at temperatures of 298, 318, 335, and 355 K. The FC-770 liquid is thermally and chemically stable and compatible with all the perovskites studied in this work. Furthermore, it evaporates without leaving residuals on the sample. emperature control is obtained with an Oxford Cryosystems cooler,[3] where the temperature offset caused by the large diameter sapphire capillary was calibrated using the diffraction pattern of silver.[4] The Dectris Pilatus 2 M detector was used for recording 2D diffraction images and the local program Bubble was used for integration of the 2D images.[1]

Temperature-dependent, lab-based X-Ray diffraction

The additional temperature-dependent X-ray diffractograms were collected using a Bruker Axs D8 Phaser Advanced. Equipped with a Cu $K_{\alpha1,2}$ (λ = 1.54184 Å) radiation source operating at 40 kV and 40 mA and an Anton Paar XRK900 Temperature Chamber. The sample height for all experiments was aligned so that the (400) reflection of $Cs_2AgBiBr_6$ was detected at 31.7°. The temperature was calibrated by measuring the lattice expansivity of MgO. For $CsPbBr_3$ the additional diffractograms were collected from 330 K to 500 K (and back) with a temperature increment of 1 K. The additional elpasolite compositions were studied between 325 K and 450 K as discussed in See **Supporting Note 4**.

Rietveld Refinement method

The refinements using the Rietveld method was performed using the FullProfSuite software. All diffractograms were fitted using Pseudo–Voigt functions with the unit cell dimensions, reflection intensity, atomic coordinates of the inorganic elements as variables. In some cases the Debye–Waller factor and the profile shape were used as variables to improve the quality of the fits. Note that including these variables only lower the residuals, without changing the lattice parameters obtained from the fits. The starting crystallographic information are obtained using files (CIF) retrieved from the ICSD database. Below a list of the used entries is given.

**Composition, phase(s)**	**ICSD entry/entries**
$MAPbI_3$, orthorhombic, tetragonal, cubic	428898, 238610, 7236651
$MAPbBr_3$, orthorhombic, tetragonal, cubic	268782, 268779, 268785
$MAPbCl_3$, orthorhombic, tetragonal, cubic	1469989**, 7236651
$MAPb(Br_{0.30}I_{0.70})_3$	243598**
$MAPb(Br_{0.70}I_{0.30})_3$	243598**
$MAPb(Cl_{0.30}I_{0.70})_3$	243598**
$MAPb(Cl_{0.70}I_{0.30})_3$	243598**
$CsPbBr_3$, orthorhombic, tetragonal, cubic	14608, 14610
$CsPbCl_3$, orthorhombic, cubic	230496, 249888, 29072
$Cs_2AgBi(Br_{0.33}I_{0.67})_6$, cubic	252164**
$Cs_2AgBiBr_6$, cubic	252164
$Cs_2AgBiCl_6$, cubic	252451
$Cs_2AgInCl_6$, cubic	257115
$Cs_2Ag(Bi_{0.5}In_{0.5})Br_6$, cubic	252164**
$Cs_2Ag(Bi_{0.5}Sb_{0.5})Br_6$, cubic	252164**
$Cs_2Ag(Bi_{0.9}Fe_{0.1})Br_6$, cubic	252164**

** For these compositions, no structural files (.cif) were available. In these cases we have used cif's of identical structures and space groups to refine the lattice parameters. For $MAPbCl_3$, we have utilized the structural file of cubic $MAPbI_3$ (ICSD 7236651). For the MA-based mixed halide compositions, we have used the structural file of $MAPb(Br_{0.15}I_{0.85})_3$ (ICSD 243598). For elpasolites with mixed trivalent cations, we have utilized the structural file of cubic $Cs_2AgBiBr_6$ (ICSD 252164). Here, we assumed stoichiometric occupancy on the metal site, which may introduce some discrepancies in fitting the relative peak heights.

Supporting Figures

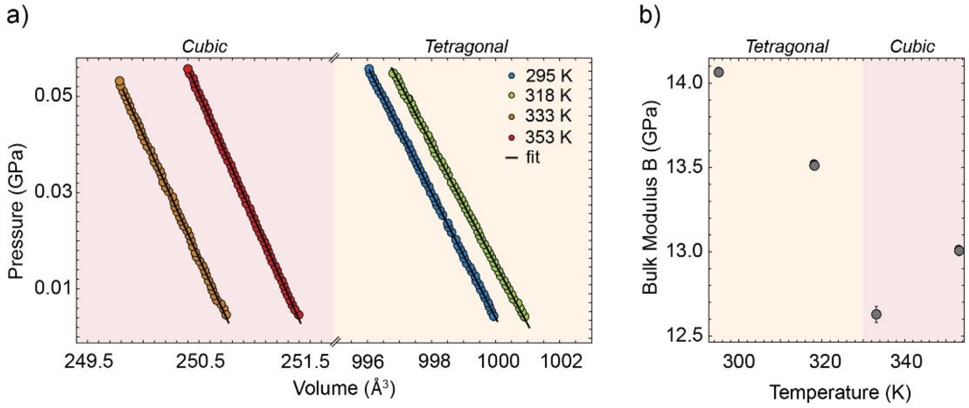

Figure S1. a) Unit-cell volume changes with pressure for $MAPbI_3$ in the tetragonal *I*4/*mcm* (yellow) and cubic *Pm*-3*m* (red) phase and the corresponding fit to equation 1 of the main text. **b)** Bulk moduli obtained from the data shown in (a) and reported as a function of temperature in the tetragonal (yellow) and cubic (red) phase. The lattice parameters versus pressure are shown in **Figure 1** of the main text.

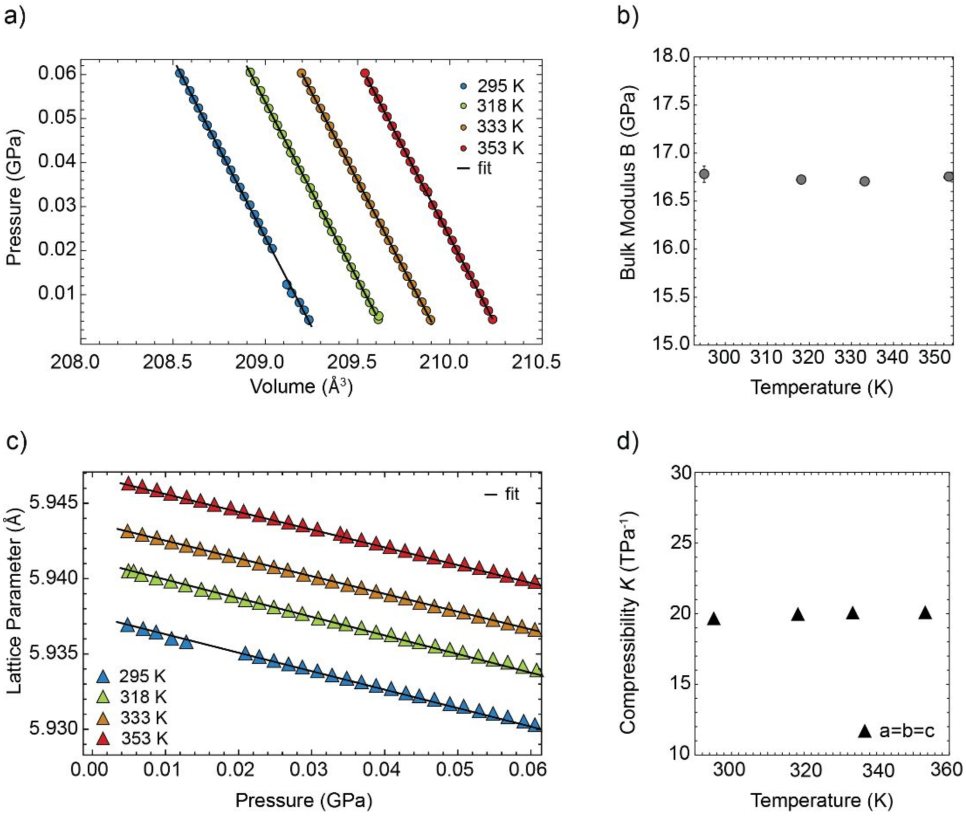

Figure S2. Unit-cell volume changes with pressure for $MAPbBr_3$ in the cubic *Pm*-3*m* phase and the corresponding fit to equation 1 of the main text. **b)** Bulk moduli obtained from the data shown in (a) and reported as a function of temperature in the cubic phase show no temperature dependence. **c)** Pressure-dependent lattice parameters of cubic $MAPbBr_3$ (Pm-3m space group) collected at 295, 318, 333, and 353 K obtained from refinements of synchrotron XRD pattern using the Rietveld method. **d)** Compressibility *K* of the lattice parameters in the cubic phase. Given the cubic structure, the compressibility is equal along all three axes.

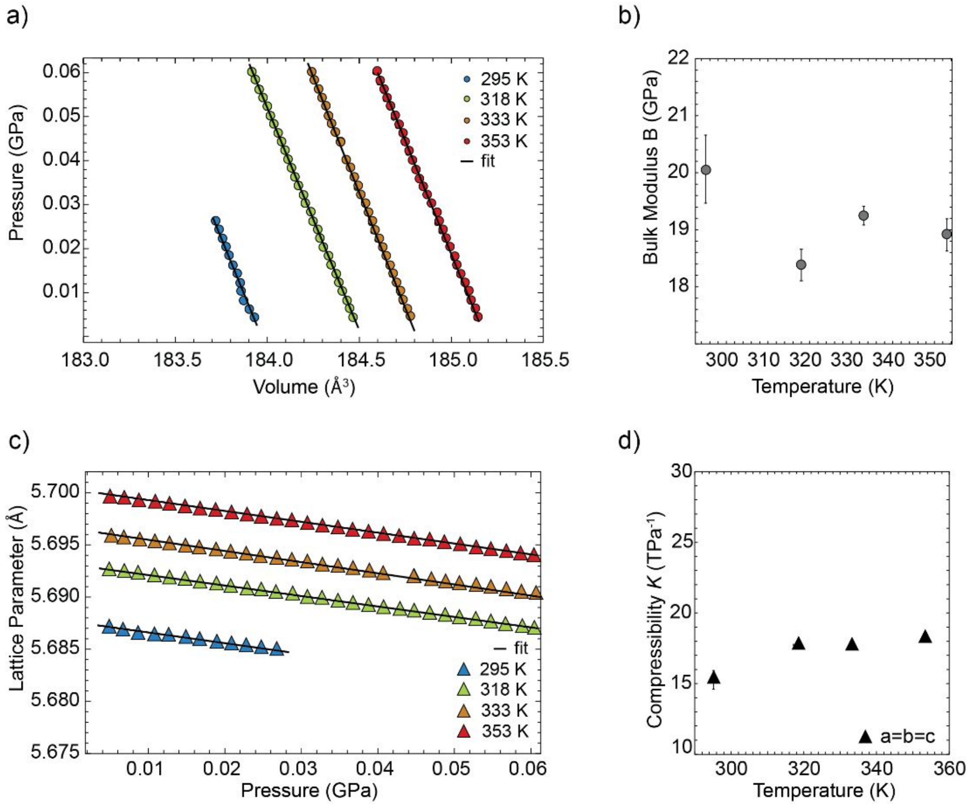

Figure S3. Unit-cell volume changes with pressure for $MAPbCl_3$ in the cubic *Pm*-3*m* phase and the corresponding fit to equation 1 of the main text. **b)** Bulk moduli obtained from the data shown in (a) and reported as a function of temperature in the cubic phase show no temperature dependence. **c)** Pressure-dependent lattice parameters of cubic $MAPbCl_3$ (Pm-3m space group) collected at 295, 318, 333, and 353 K obtained from refinements of synchrotron XRD pattern using the Rietveld method. **d)** Compressibility *K* of the lattice parameters in the cubic phase. Given the cubic structure, the compressibility is equal along all three axes.

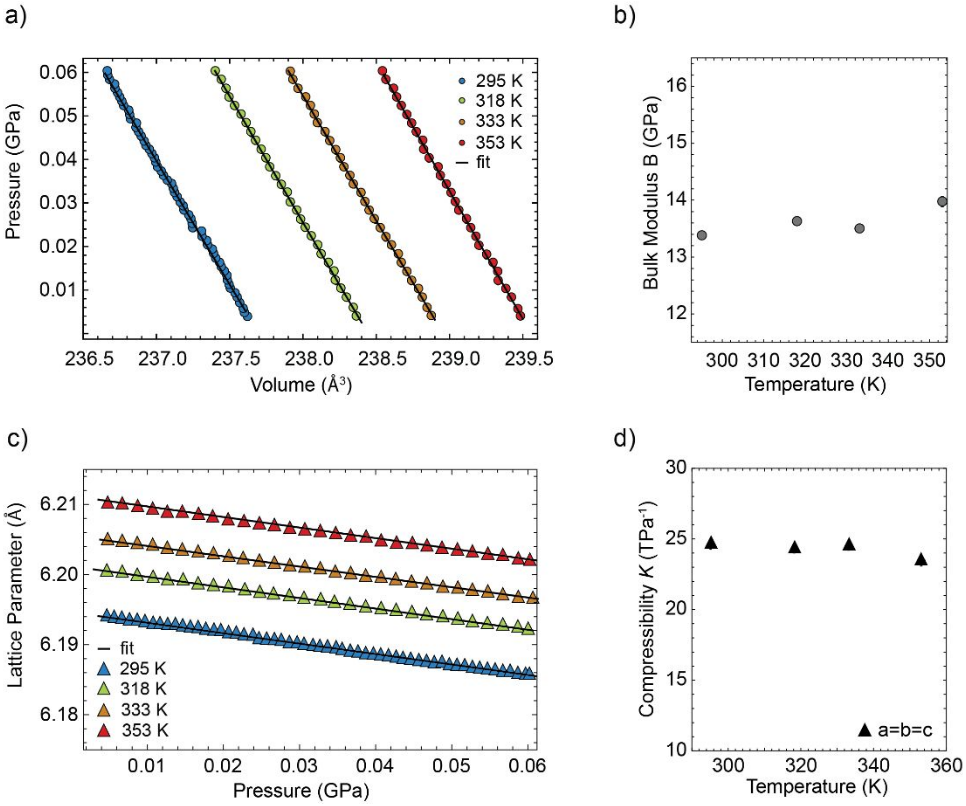

Figure S4. Unit-cell volume changes with pressure for $MAPb(Br_{0.3}I_{0.7})_3$ in the cubic *Pm*-3*m* phase and the corresponding fit to equation 1 of the main text. **b)** Bulk moduli obtained from the data shown in (a) and reported as a function of temperature in the cubic phase show no temperature dependence. **c)** Pressure-dependent lattice parameters of cubic $MAPb(Br_{0.3}I_{0.7})_3$ (Pm-3m space group) collected at 295, 318, 333, and 353 K obtained from refinements of synchrotron XRD pattern using the Rietveld method. **d)** Compressibility *K* of the lattice parameters in the cubic phase. Given the cubic structure, the compressibility is equal along all three axes.

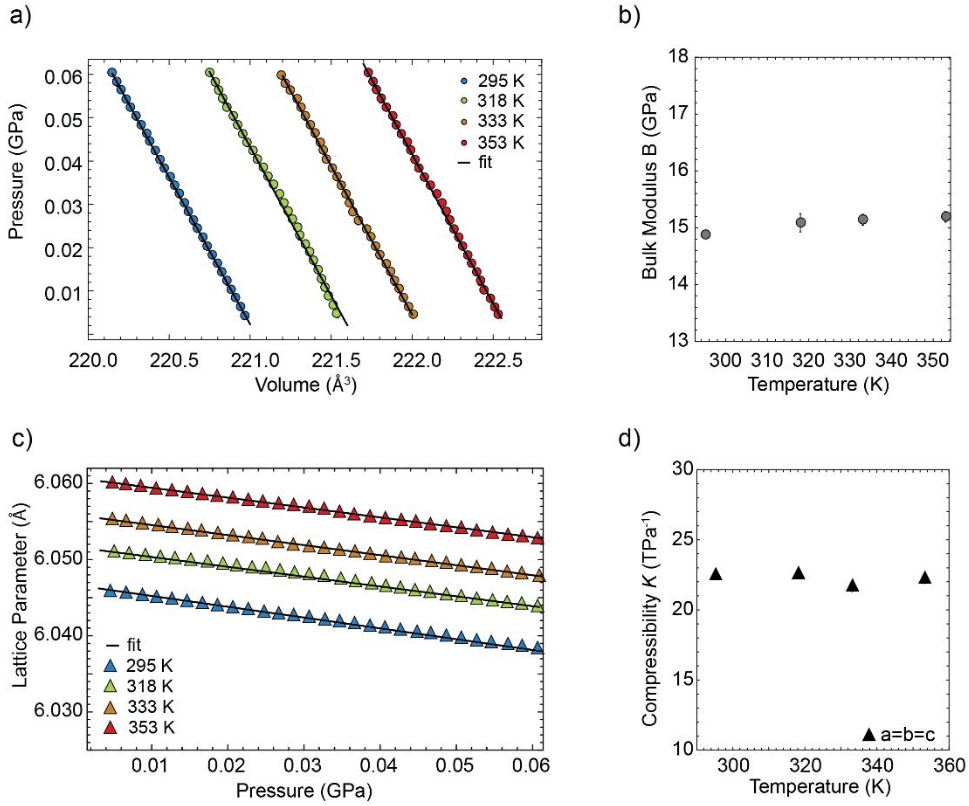

Figure S5. Unit-cell volume changes with pressure for $MAPb(Br_{0.7}I_{0.3})_3$ in the cubic *Pm*-3*m* phase and the corresponding fit to equation 1 of the main text. **b)** Bulk moduli obtained from the data shown in (a) and reported as a function of temperature in the cubic phase show no temperature dependence. **c)** Pressure-dependent lattice parameters of cubic $MAPb(Br_{0.7}I_{0.3})_3$ (Pm-3m space group) collected at 295, 318, 333, and 353 K obtained from refinements of synchrotron XRD pattern using the Rietveld method. **d)** Compressibility *K* of the lattice parameters in the cubic phase. Given the cubic structure, the compressibility is equal along all three axes.

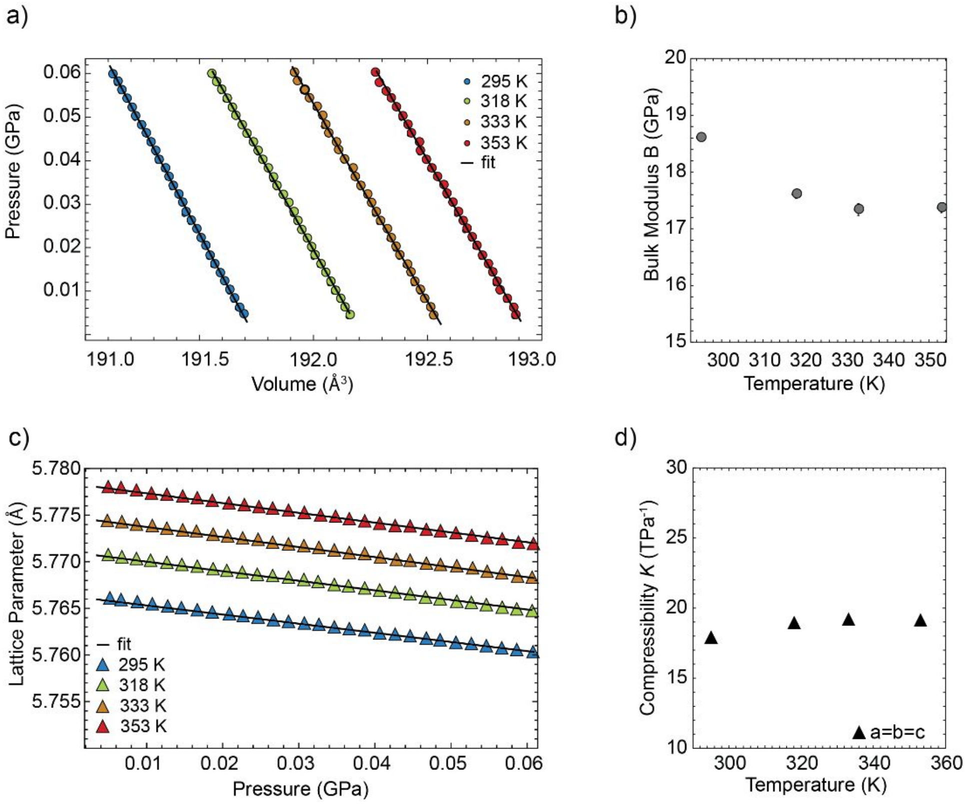

Figure S6. Unit-cell volume changes with pressure for $MAPb(Br_{0.3}Cl_{0.7})_3$ in the cubic *Pm*-3*m* phase and the corresponding fit to equation 1 of the main text. **b)** Bulk moduli obtained from the data shown in (a) and reported as a function of temperature in the cubic phase show no temperature dependence. **c)** Pressure-dependent lattice parameters of cubic $MAPb(Br_{0.3}Cl_{0.7})_3$ (Pm-3m space group) collected at 295, 318, 333, and 353 K obtained from refinements of synchrotron XRD pattern using the Rietveld method. **d)** Compressibility *K* of the lattice parameters in the cubic phase. Given the cubic structure, the compressibility is equal along all three axes.

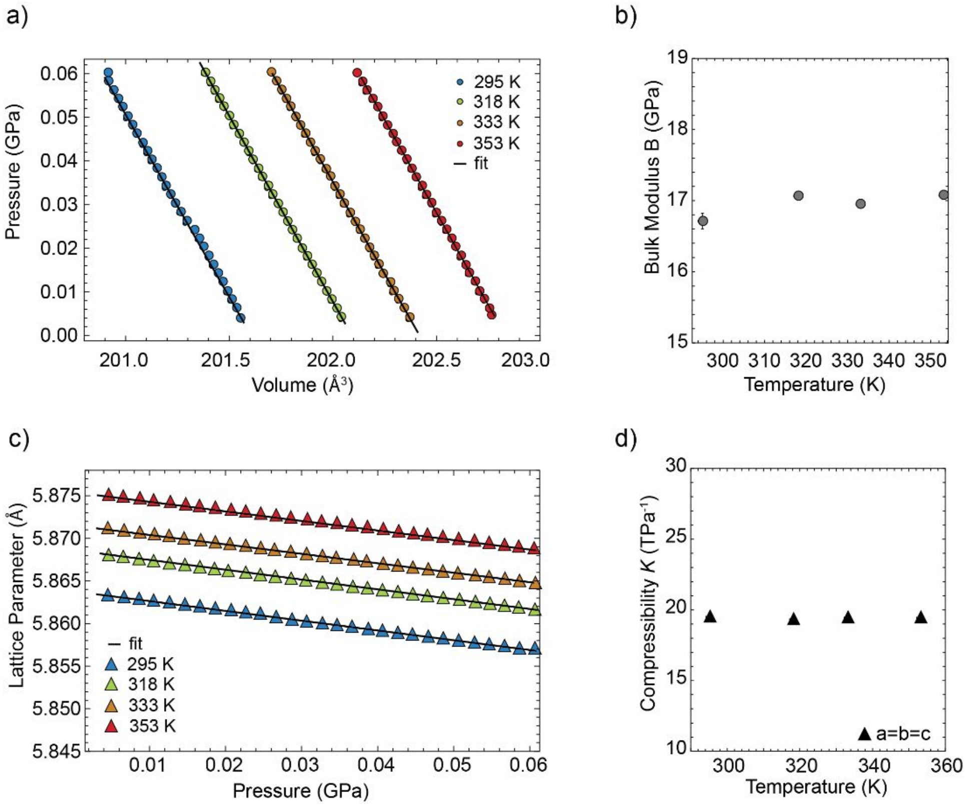

Figure S7. Unit-cell volume changes with pressure for $MAPb(Br_{0.7}Cl_{0.3})_3$ in the cubic *Pm*-3*m* phase and the corresponding fit to equation 1 of the main text. **b)** Bulk moduli obtained from the data shown in (a) and reported as a function of temperature in the cubic phase show no temperature dependence. **c)** Pressure-dependent lattice parameters of cubic $MAPb(Br_{0.7}Cl_{0.3})_3$ (Pm-3m space group) collected at 295, 318, 333, and 353 K obtained from refinements of synchrotron XRD pattern using the Rietveld method. **d)** Compressibility *K* of the lattice parameters in the cubic phase. Given the cubic structure, the compressibility is equal along all three axes.

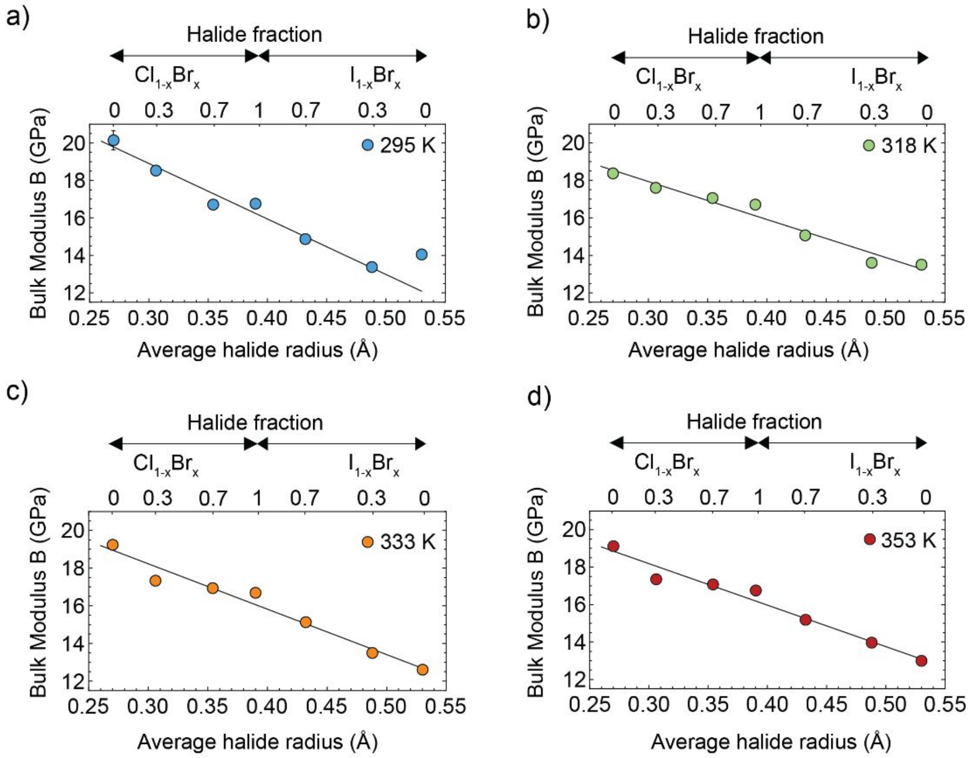

Figure S8. Linear fit of the bulk modulus as a function of the average halide radius at **a)** 295 K, **b)** 318 K, **c)** 333 K and **d)** 353K for single halide and mixed-halide compositions. Bulk moduli of compositions with intermediate average halide radius can be calculated using $B = 25.9 - 24.1x$ at room temperature, $B = 24 - 20.2x$ at 318K, $B = 25.4 - 24.1x$ at 333K, $B = 24.8 - 22.1x$ at 353K, where B is the bulk modulus and x the average halide radius.

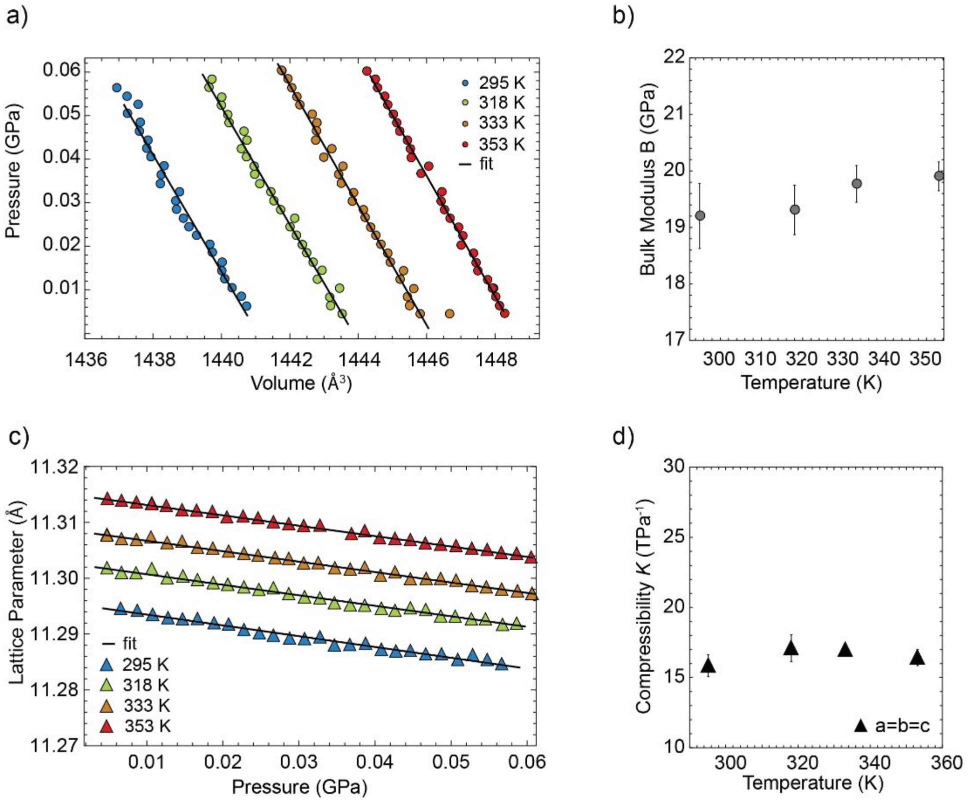

Figure S9. Unit-cell volume changes with pressure for $Cs_2AgBiBr_6$ in the cubic *Fm*-3*m* phase and the corresponding fit to equation 1 of the main text. **b)** Bulk moduli obtained from the data shown in (a) and reported as a function of temperature in the cubic phase show no temperature dependence. **c)** Pressure-dependent lattice parameters of cubic $Cs_2AgBiBr_6$ (*Fm*-3*m* space group) collected at 295, 318, 333, and 353 K obtained from refinements of synchrotron XRD pattern using the Rietveld method. **d)** Compressibility *K* of the lattice parameters in the cubic phase. Given the cubic structure, the compressibility is equal along all three axes.

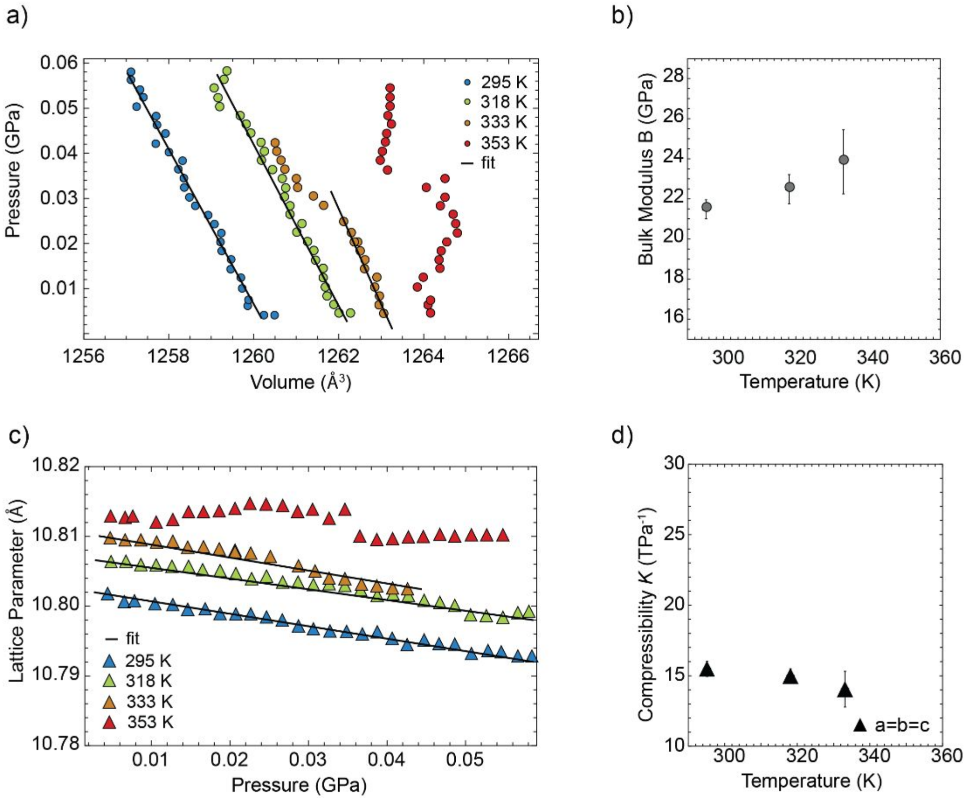

Figure S10. Unit-cell volume changes with pressure for $Cs_2AgBiCl_6$ in the cubic *Fm*-3*m* phase and the corresponding fit to equation 1 of the main text. **b)** Bulk moduli obtained from the data shown in (a) and reported as a function of temperature in the cubic phase show no temperature dependence. Due to the deviation in the linearity of P-V curve, we only fit the first half of the curve at 333 K, whereas we are not able to fit the P-V curve at 353 K. **c)** Pressure-dependent lattice parameters of cubic $Cs_2AgBiCl_6$ (*Fm*-3*m* space group) collected at 295, 318, 333, and 353 K obtained from refinements of synchrotron XRD pattern using the Rietveld method. **d)** Compressibility *K* of the lattice parameters in the cubic phase. Given the cubic structure, the compressibility is equal along all the three axes. Due to the deviation in the linearity of P-V curve at 353 K we do not report the corresponding compressibility.

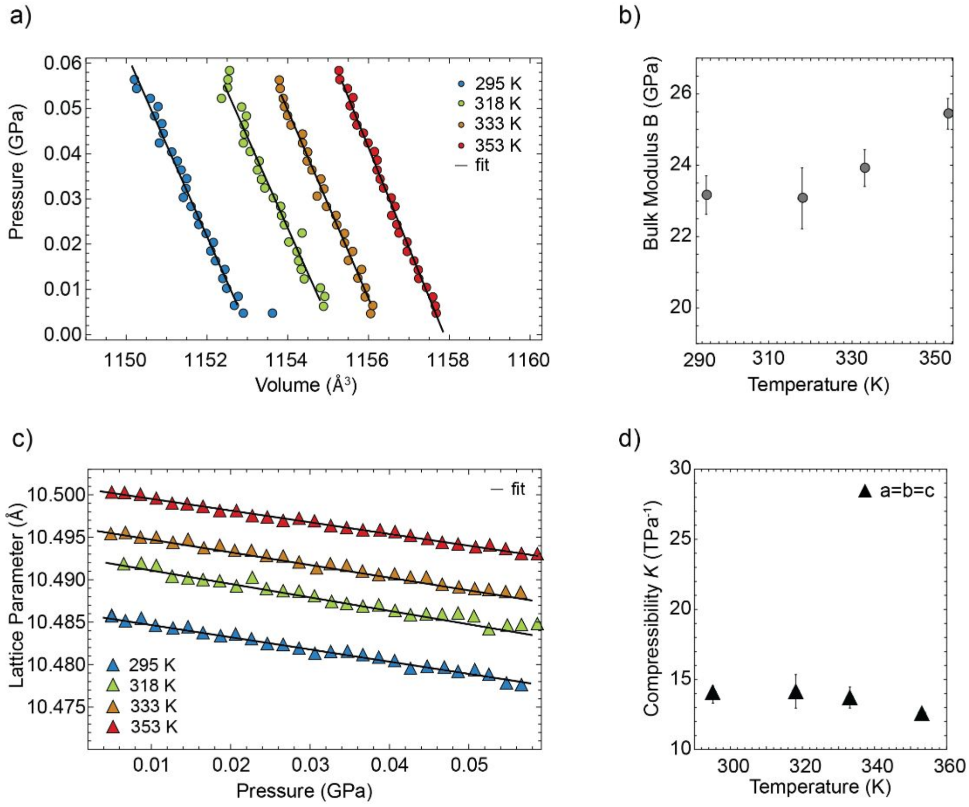

Figure S11. Unit-cell volume changes with pressure for $Cs_2AgInCl_6$ in the cubic *Fm*-3*m* phase and the corresponding fit to equation 1 of the main text. **b)** Bulk moduli obtained from the data shown in (a) and reported as a function of temperature in the cubic phase show no temperature dependence. **c)** Pressure-dependent lattice parameters of cubic $Cs_2AgInCl_6$ (*Fm*-3*m* space group) collected at 295, 318, 333, and 353 K obtained from refinements of synchrotron XRD pattern using the Rietveld method. **d)** Compressibility *K* of the lattice parameters in the cubic phase. Given the cubic structure, the compressibility is equal along all three axes.

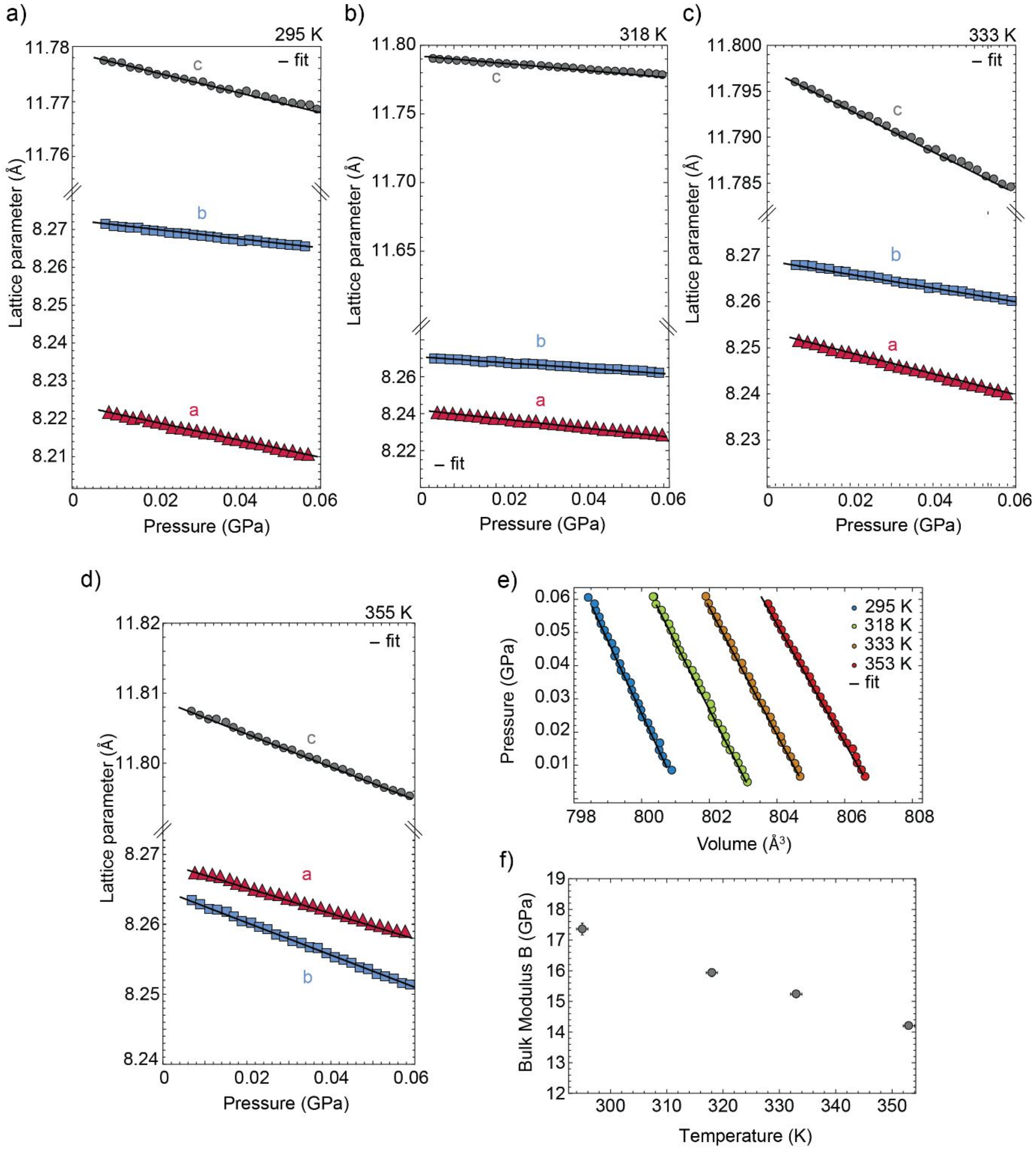

Figure S12. Pressure-dependent lattice parameters of the orthorhombic $CsPbBr_3$ (*Pbnm* space group) obtained from refinements of synchrotron XRD pattern at **a)** 295 K, **b)** 318 K, **c)** 333 K and **d)** 355 K using the Rietveld method. At 355 K, the lattice parameter *a* swap with that of *b*. **e)** Unit-cell volume changes with pressure for $CsPbBr_3$ in the orthorhombic *Pbnm* phase. **f)** Bulk modulus of $CsPbBr_3$ as a function of temperature obtained from the fit of the volume changes with pressure to equation 1 of the main text. Compressibility *K* is shown in **Figure 3b** in the main text.

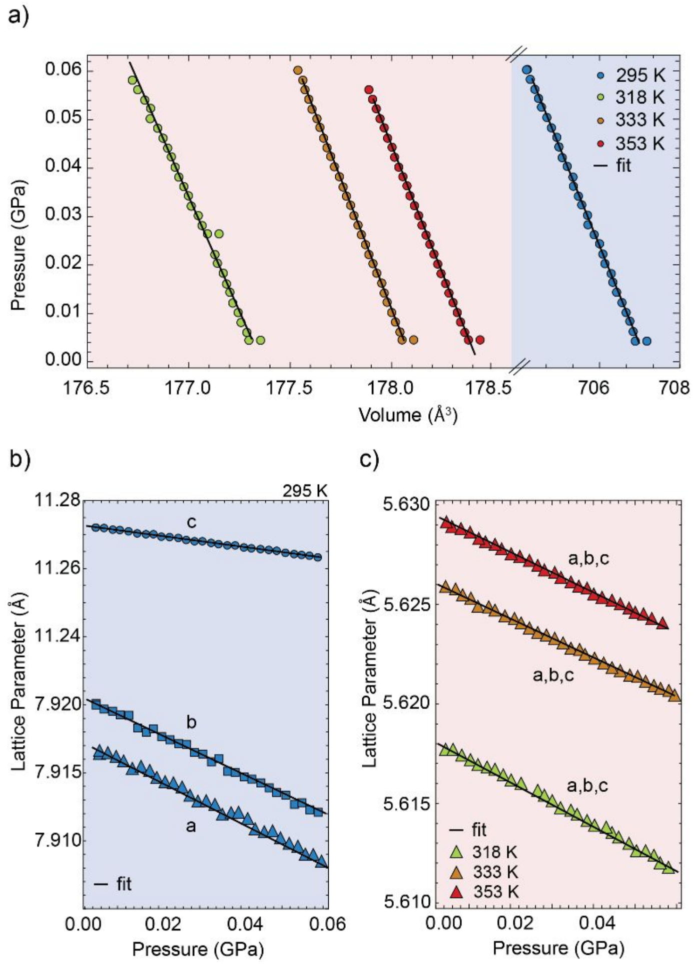

Figure S13. a) Unit-cell volume changes with pressure for $CsPbCl_3$ in the cubic *Pm*-3*m* phase (highlighted in red) and in the orthorhombic *Pbnm* phase (highlighted in blue) and the corresponding fit to equation 1 of the main text. **b)** Pressure-dependent lattice parameters of the orthorhombic $CsPbCl_3$ (*Pbnm* space group) collected at 295 K and **c)** of cubic Pm-3m phase at 318, 333, and 353 K obtained from refinements of synchrotron XRD pattern using the Rietveld method. Compressibility *K* is shown in **Figure 3b** in the main text.

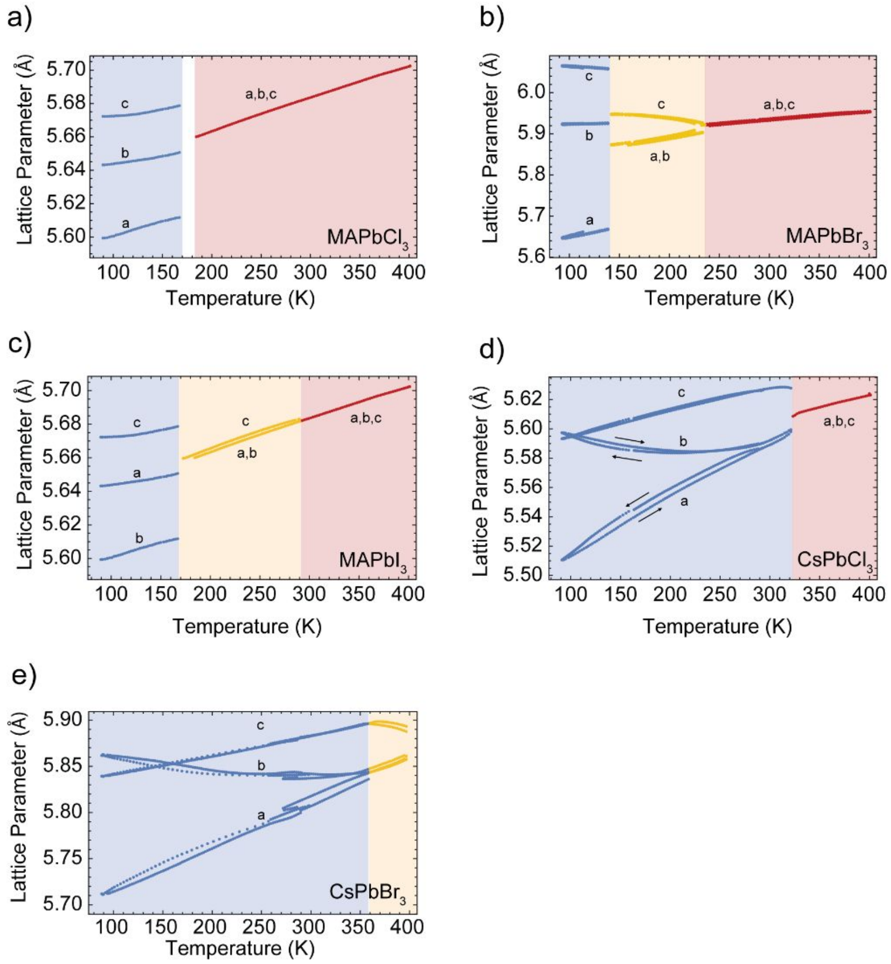

Figure S14. Pseudocubic lattice parameters of **a)** $MAPbCl_3$, **b)** $MAPbBr_3$, **c)** $MAPbI_3$, **d)** $CsPbCl_3$, **e)** $CsPbBr_3$ between 90 K and 400 K. The blue, yellow and red regions correspond to the orthorhombic, tetragonal and cubic phase, respectively. The regions in white correspond to the temperature range where two phases coexist in the XRD patterns. The black arrows indicate the direction of temperature variation, *i.e.* cooling (arrow down) and heating (arrow up). Solid lines represent the fit for the linear thermal expansivity.

Supporting Note 1. Fit for determining the bulk modulus as a function of temperature

For all the elpasolite, single halide (iodide, bromide, chloride) and mixed-halide perovskite series $MAPb(Cl_{1-x}Br_x)_3$ and $MAPb(I_{1-x}Br_x)_3$, we estimate the bulk moduli (B) by fitting the following function to the pressure-volume trends (**Figure 1c–d** and **Figure S1–S7**) at 295, 318, 333 and 353K.

$$B = -\frac{\Delta P}{\Delta V}V \tag{2}$$

with $\Delta P/\Delta V$ the partial derivative of pressure with respect to volume, and V the volume at ambient pressure. The results are reported below in **Table S1**.

	B **(GPa) at 295 K**	***B*** **(GPa) at 318 K**	***B*** **(GPa) at 333 K**	***B*** **(GPa) at 353 K**
$\mathbf{MAPbI_3}$	14.22(2)	13.67(3)	12.77(4)	13.15(3)
$\mathbf{MAPbBr_3}$	16.94(8)	16.87(4)	16.85(3)	16.91(3)
$\mathbf{MAPbCl_3}$	20.09(91)	18.78(7)	19.07(5)	18.58(7)
$\mathbf{MAPb(Br_{0.3}I_{0.7})_3}$	13.53(7)	13.78(6)	13.66(6)	14.13(9)
$\mathbf{MAPb(Br_{0.7}I_{0.3})_3}$	15.03(4)	15.25(15)	15.30(9)	15.36(9)
$\mathbf{MAPb(Br_{0.3}Cl_{0.7})_3}$	18.77(6)	17.77(7)	17.50(10)	17.53(8)
$\mathbf{MAPb(Br_{0.7}Cl_{0.3})_3}$	16.87(11)	17.22(2)	17.11(4)	17.24(4)
$\mathbf{CsPbBr_3}$	17.53(19)	16.10(12)	15.40(8)	14.36(7)
$\mathbf{CsPbCl_3}$	19.07(14)	16.82(16)	19.16(5)	18.79(5)
$\mathbf{Cs_2AgBiBr_6}$	19.35(57)	19.46(43)	19.93(32)	20.06(25)
$\mathbf{Cs_2AgBiCl_6}$	21.63(47)	22.65(72)	15.92(79)	-
$\mathbf{Cs_2AgInCl_6}$	23.31(53)	23.23(85)	24.07(51)	25.59(43)

Table S1. Bulk moduli, B, as a function of temperature for all compositions at 295 K, 318 K, 333 K, and 353 K.

Supporting Note 2. Axes-dependent and volumetric compressibility as a function of temperature

Considering that the elastic properties of non-cubic perovskites, such as tetragonal $MAPbI_3$, are in general anisotropic and therefore depend on crystallographic direction, we derived the compressibility of the specific crystal axes by monitoring their compression as a function of applied pressure (*e.g.*, for the compressibility along a-axis):

$$K_a = -\frac{(a_f - a_0)}{a_f}P \tag{3}$$

with a the lattice parameter, and $a_f - a_0$ the change in lattice parameter between the lattice parameter at the final (a_f) and initial (a_0) pressure. Similarly, the compressibility for b- and c-axis, for non-cubic perovskites, can be determined. The results are reported in **Table S2-S4**. The volume compressibility, K_V, i.e., the susceptibility of a material to compress upon external applied pressure, is inversely proportional to B. K_V is given by

$$K_V = K_a + K_b + K_c \tag{4}$$

i.e., K_V is the sum of the compressibility along each axis. Results are reported in **Table S5**.

		K_x (TPa^{-1}) at 295 K		K_x (TPa^{-1}) at 318 K		K_x (TPa^{-1}) at 333 K		K_x (TPa^{-1}) at 353 K
**$CsPbBr_3$**	a	27.62(29)	a	27.52(20)	a	27.78(16)	a	21.44(18)
	b	13.85(22)	b	16.95(20)	b	18.65(16)	b	28.48(21)
	c	15.72(23)	c	17.75(16)	c	18.62(13)	c	19.85(13)
**$CsPbCl_3$**	a	19.15(33)		-		-		-
	b	19.06(26)						
	c	14.45(14)						

Table S2. Compressibility along the a, b, and c-axis, K_x $(with\ x = a, b, c)$, for the orthorhombic compositions at 295 K, 318 K, 333 K, and 353 K.

	K_x (TPa^{-1}) at 295 K		K_x (TPa^{-1}) at 318 K		K_x (TPa^{-1}) at 333 K	K_x (TPa^{-1}) at 353 K
**$MAPbI_3$**	$a = b$	28.25(5)	$a = b$	28.85(9)	-	-
	c	13.89(5)	c	15.60(9)		

Table S3. Compressibility along the a, b, and c-axis, K_x $(with\ x = a,b,c)$, for the tetragonal compositions at 295 K, 318 K, 333 K, and 353 K.

	K_a (TPa^{-1}) at 295 K	K_a (TPa^{-1}) at 318 K	K_a (TPa^{-1}) at 333 K	K_a (TPa^{-1}) at 353 K
**$MAPbI_3$**	-	-	26.14(9)	25.38(5)
**$MAPbBr_3$**	19.68(9)	19.78(5)	19.76(4)	19.73(4)
**$MAPbCl_3$**	12.05(39)	17.76(6)	17.50(5)	17.96(7)
**$MAPb(Br_{0.3}I_{0.7})_3$**	24.65(12)	24.17(11)	24.44(10)	23.61(15)
**$MAPb(Br_{0.7}I_{0.3})_3$**	22.20(5)	21.87(22)	21.81(13)	21.73(12)
**$MAPb(Br_{0.3}Cl_{0.7})_3$**	17.78(5)	18.76(8)	19.06(11)	19.03(9)
**$MAPb(Br_{0.7}Cl_{0.3})_3$**	19.78(13)	19.37(2)	19.50(4)	19.33(4)
**$CsPbCl_3$**	-	17.83(20)	17.41(5)	17.76(5)
**$Cs_2AgBiBr_6$**	17.24(51)	17.14(38)	16.74(27)	16.63(21)
**$Cs_2AgBiCl_6$**	15.42(33)	14.73(47)	20.96(104)	15.96(342)
**$Cs_2AgInCl_6$**	14.31(32)	14.36(52)	13.86(29)	13.03(22)

Table S4. Compressibility along the a-axis, K_a, for the cubic compositions at 295 K, 318 K, 333 K, and 353 K.

	K_V (TPa^{-1}) at 295 K	K_V (TPa^{-1}) at 318 K	K_V (TPa^{-1}) at 333 K	K_V (TPa^{-1}) at 353 K
**$MAPbI_3$**	70.30(12)	73.15(15)	78.32(29)	76.03(17)
**$MAPbBr_3$**	59.02(29)	59.26(16)	59.34(10)	59.13(11)
**$MAPbCl_3$**	36.14(119)	53.24(20)	52.42(15)	53.82(21)
**$MAPb(Br_{0.3}I_{0.7})_3$**	73.86(38)	72.56(33)	73.20(32)	70.74(46)
**$MAPb(Br_{0.7}I_{0.3})_3$**	66.50(16)	65.55(66)	65.36(42)	65.12(38)
**$MAPb(Br_{0.3}Cl_{0.7})_3$**	53.27(17)	56.26(24)	57.13(33)	57.03(27)
**$MAPb(Br_{0.7}Cl_{0.3})_3$**	59.28(39)	58.05(8)	58.44(14)	58.02(14)
**$CsPbBr_3$**	57.05(63)	62.10(48)	64.93(37)	69.63(38)
**$CsPbCl_3$**	52.43(40)	59.42(59)	52.17(15)	53.22(14)
**$Cs_2AgBiBr_6$**	51.68(154)	51.38(115)	50.17(81)	49.84(63)
**$Cs_2AgBiCl_6$**	46.23(100)	44.15(141)	62.82(313)	-
**$Cs_2AgInCl_6$**	42.89(98)	43.05(1.58)	41.54(89)	39.07(65)

Table S5. Volumetric compressibility K_V for the compositions studied at 295 K, 318 K, 333 K, and 353 K.

Supporting Note 3. Calculation of the thermal expansivity

The thermal expansion coefficient, α_L, of a material is defined as the relative expansion in one dimension per unit of temperature:

$$\alpha_L = \frac{1\Delta L}{L\Delta T} \tag{5}$$

For solids α_L is typically temperature-independent. So, we expect a linear dependence of the lattice parameters, $L = a$, b, and c, over our range of measured temperatures. As such for $L = a$:

$$\alpha_\mathrm{a} = \frac{1\Delta a}{a\Delta T} \tag{6}$$

Rewriting eq. (6) provides:

$$\frac{\Delta a}{a} = \alpha_\mathrm{a}\Delta T \tag{7}$$

For an α_a that is independent to temperature this yields:

$$a(T) = a_0 + \alpha_\mathrm{a} a_0 T \tag{8}$$

where a_0 is the lattice parameter extrapolated to T = 0 K. For some compositions we observe non-linear expansion along certain crystal axes. In this case a single value for α_a does not suffice and integration of eq. (6) over the experimental temperature range is required:

$$a(T) = a_0 Exp\left[\int_{T_1}^{T_2} \alpha_\mathrm{a}(T)dT\right] \tag{9}$$

with a(T) a second order polynomial function ($a(T) = m_1T^2 + m_2T + a_0$) describing the lattice parameter as a function of temperature. Solving eq. (9) for α_a gives:

$$\alpha_\mathrm{a}(T) = \frac{m_2 + 2m_1T}{a_0 + T(m_2 + m_1T)} \tag{10}$$

Table S6 provides the optimized parameters for eq. (10).

**i)**	**Orthorhombic**			
	Crystal axis	m_1	m_2	a_0
**$MAPbI_3$**	a ($\times 10^{-5}$ K^{-1})	3.81(12)		
	b ($\times 10^{-5}$ K^{-1})	2.02(7)		
	c ($\times 10^{-5}$ K^{-1})	5.81(10)		
**$MAPbBr_3$**	a ($\times 10^{-5}$ K^{-1})	9.04(10)		
	b ($\times 10^{-5}$ K^{-1})	−2.79(5)		
	c ($\times 10^{-5}$ K^{-1})	1.00(2)		
**$MAPbCl_3$**	a	-8.45×10^{-7}	5.63×10^{-4}	11.15
	b	1.86×10^{-6}	-3.05×10^{-4}	11.36
	C	8.60×10^{-7}	-3.26×10^{-5}	11.28
**$CsPbBr_3$**	a ($\times 10^{-5}$ K^{-1})	7.68(5)		
	b	1.22×10^{-6}	-6.14×10^{-4}	8.34
	c ($\times 10^{-5}$ K^{-1})	3.511(8)		
**$CsPbCl_3$**	a	-9.82×10^{-7}	9.18×10^{-4}	7.72
	b	-4.61×10^{-7}	5.14×10^{-4}	11.14
	c	1.41	-5.80×10^{-4}	7.96

**ii)**	**Tetragonal**			
	Crystal axis	m_1	m_2	a_0
**$MAPbI_3$**	$a = b$	$1.03(5) \times 10^{-6}$	5.10×10^{-5}	8.76
	c ($\times 10^{-5}$ K^{-1})	0.34(1)		
**$MAPbBr_3$**	$a = b$ ($\times 10^{-5}$ K^{-1})	6.99(8)		
	c	-5.52×10^{-6}	1.63×10^{-3}	11.77

**iii)**	**Cubic** ($\times 10^{-5}$ K^{-1})

**$MAPbI_3$**	4.04(3)
**$MAPbBr_3$**	3.53(1)
**$MAPbCl_3$**	3.51(8)
**$CsPbBr_3$**	3.27(1)**
**$CsPbCl_3$**	3.02(1)
**$Cs_2AgBi(Br_{0.33}I_{0.67})_6$**	3.39(14)**
**$Cs_2AgBiBr_6$**	2.86(7)
**$Cs_2AgBiCl_6$**	2.42(1)
**$Cs_2AgInCl_6$**	2.49(1)
**$Cs_2Ag(Bi_{0.5}In_{0.5})Br_6$**	2.84(4)**
**$Cs_2Ag(Bi_{0.5}Sb_{0.5})Br_6$**	3.34(3)**
**$Cs_2Ag(Bi_{0.9}Fe_{0.1})Br_6$**	2.06(2)**

Table S6. Optimized parameters to describe α(T) for a given temperature range.

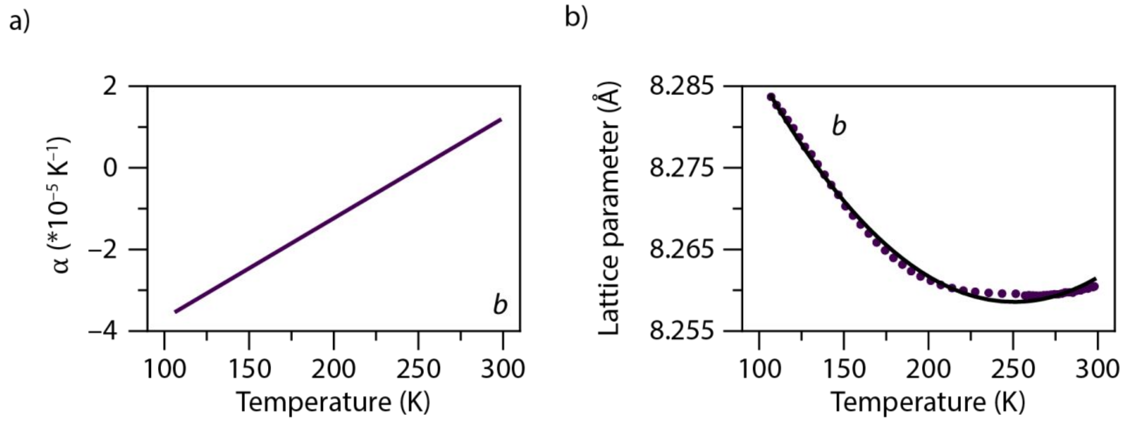

Figure S15. a) Non-linear α of the lattice parameter, b, of orthorhombic $CsPbBr_3$ between 100 and 300 K. The optimized parameters for this plot are provided in **Table S6 i)**. **b)** Temperature dependent lattice parameter of the lattice parameter, b, of orthorhombic $CsPbBr_3$ between 100 and 300 K (dots) and the polynomial fit (dotted line) to extract the temperature dependent α given in **a)**.

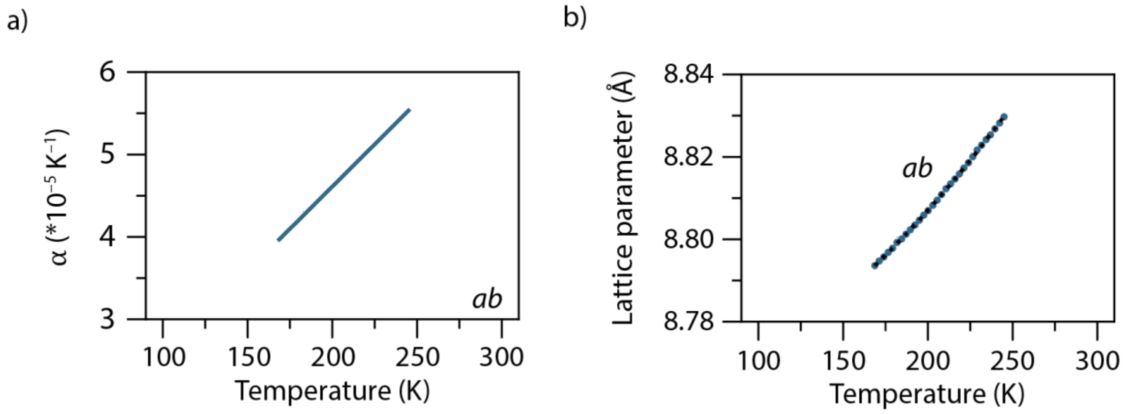

Figure S16. a) Non-linear α of the lattice parameter, *ab*, of tetragonal $MAPbI_3$ between 170 and 250 K. The optimized parameters for this plot are provided in **Table S6 ii)**. **b)** Temperature dependent lattice parameter of the lattice parameter, *ab*, of tetragonal $MAPbI_3$ between 170 and 250 K (dots) and the polynomial fit (dotted line) to extract the temperature dependent α given in **a)**.

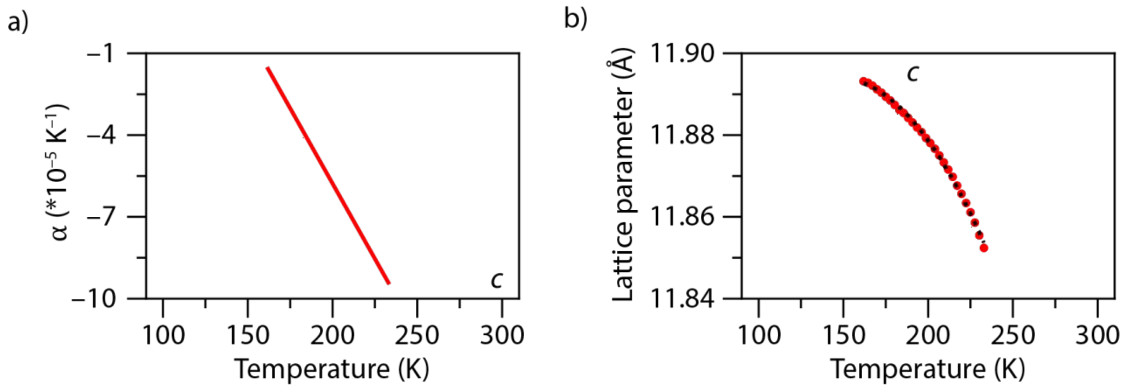

Figure S17. a) Non-linear α of the lattice parameter, *c*, of tetragonal $MAPbBr_3$ between 160 and 230 K. The optimized parameters for this plot are provided in **Table S6 ii)**. **b)** Temperature dependent lattice parameter of the lattice parameter, *c*, of tetragonal $MAPbBr_3$ between 160 and 230 K (dots) and the polynomial fit (dotted line) to extract the temperature dependent α given in **a)**.

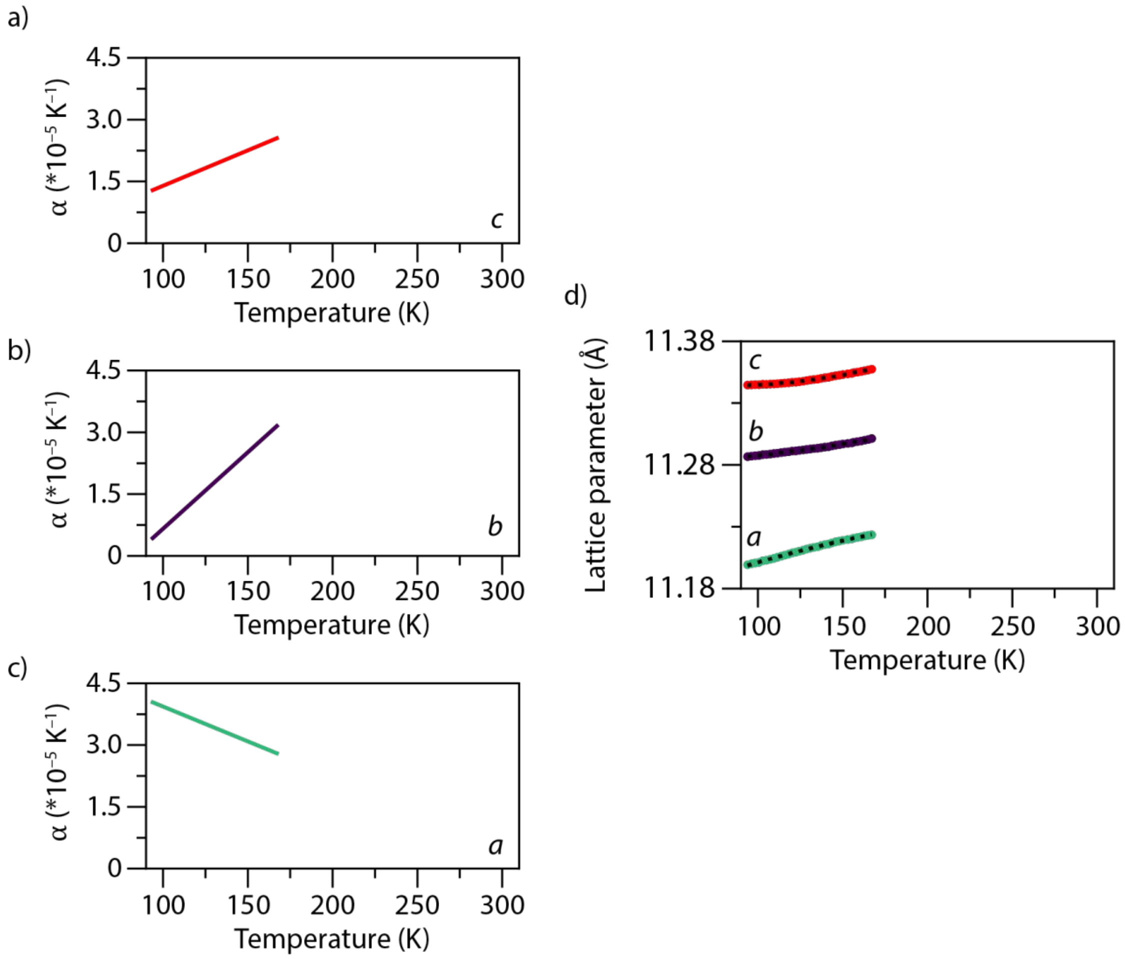

Figure S18. a–c) Non-linear α of the lattice parameters, *abc*, of orthorhombic $MAPbCl_3$ between 100 and 170 K. The optimized parameters for this plot are provided in **Table S6 i)**. **d)** Temperature dependent lattice parameter of the lattice parameter, *abc*, of orthorhombic $MAPbCl_3$ between 100 and 170 K (dots) and the polynomial fits (dotted line) to extract the temperature dependent α given in **a–c)**.

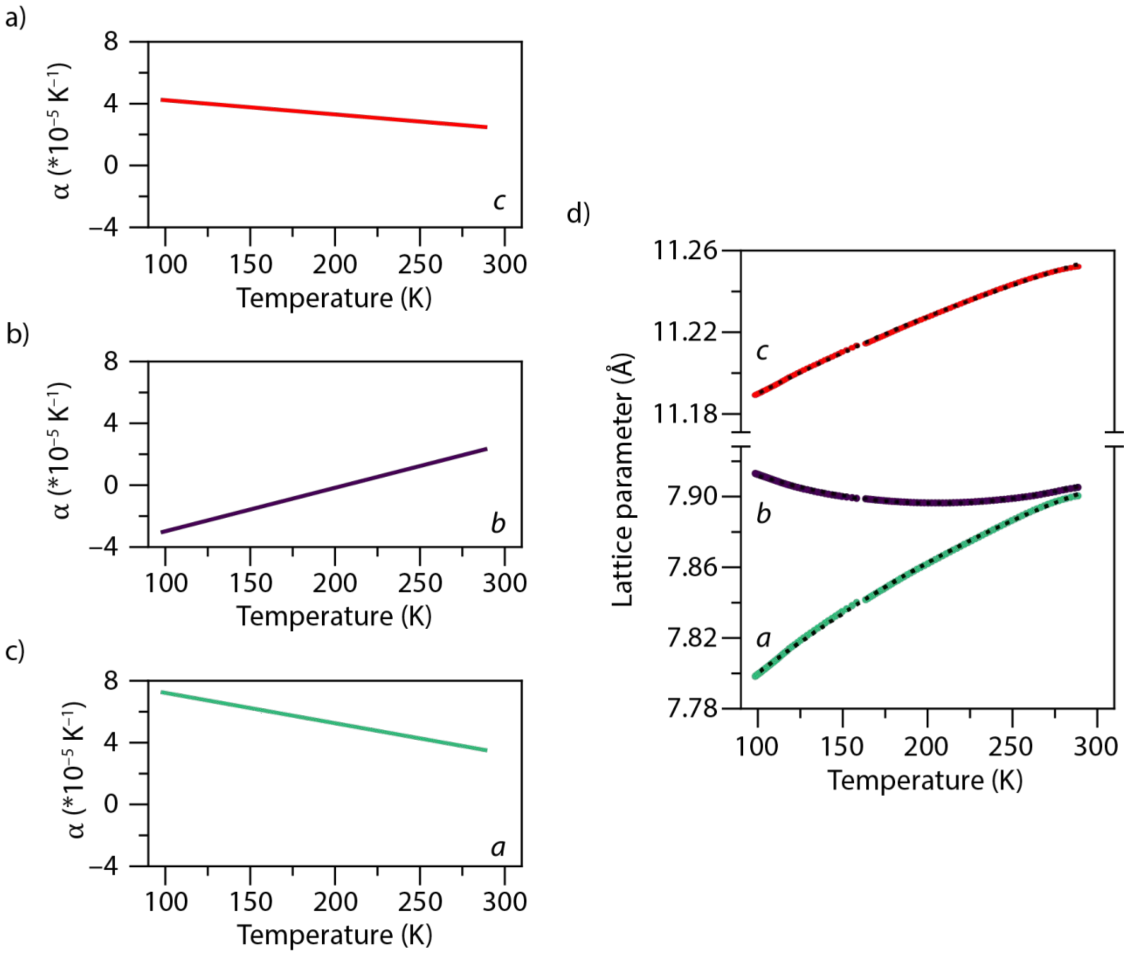

Figure S19. a–c) Non-linear α of the lattice parameters, *abc*, of orthorhombic $CsPbCl_3$ between 100 and 300 K. The optimized parameters for this plot are provided in **Table S6 i)**. **d)** Temperature dependent lattice parameter of the lattice parameter, *abc*, of orthorhombic $CsPbCl_3$ between 100 and 300 K (dots) and the polynomial fits (dotted line) to extract the temperature dependent α given in **a–c)**.

Supporting Note 4. Lab-based temperature dependent X-ray diffraction

First the temperature inside the cell was calibrated by measuring the lattice expansion of MgO over a temperature range of 300 K to 900 K. From literature the lattice parameter as a function of temperature is given by[5]:

$$a(T) = 4.2094 + 5.92 \times 10^{-5} \times T \quad (1)$$

with T the temperature in degrees Celsius. The lattice parameter of MgO as a function of temperature was determined using the same refinement method as described in the main text (**Figure S20a**). The experimental temperature was scaled with a factor of 1.05 to match the α of MgO reported in the literature (eq. (1)). No correction was performed to adjust for the change in sample position as a consequence of the expanding sample holder upon increasing the temperature.

To verify the reproducibility of our experiments, the α of $Cs_2AgBiBr_6$ (**Figure S20**, blue data points) and $CsPbBr_3$ (**Figure S21**) was determined and compared to the results from our experiments using synchrotron radiation. The discrepancy observed for these values can be attributed when considering the different experimental setups and conditions, such as the (mono-chromatic) light source, less accurately defined sample position, used detector, atmosphere, etc. We, however, note that quality of the lab-based data is still sufficient to apply the same refinement method as described in the main text. As such, we determined the α of $Cs_2Ag(Bi_{1-x}B_x)Br_6$ with (B_x = $In_{0.5}$, $Sb_{0.5}$ and $Fe_{0.1}$) and $Cs_2AgBi(Br_{0.33}I_{0.67})_6$ (**Figure S20b** and **Table 1**).

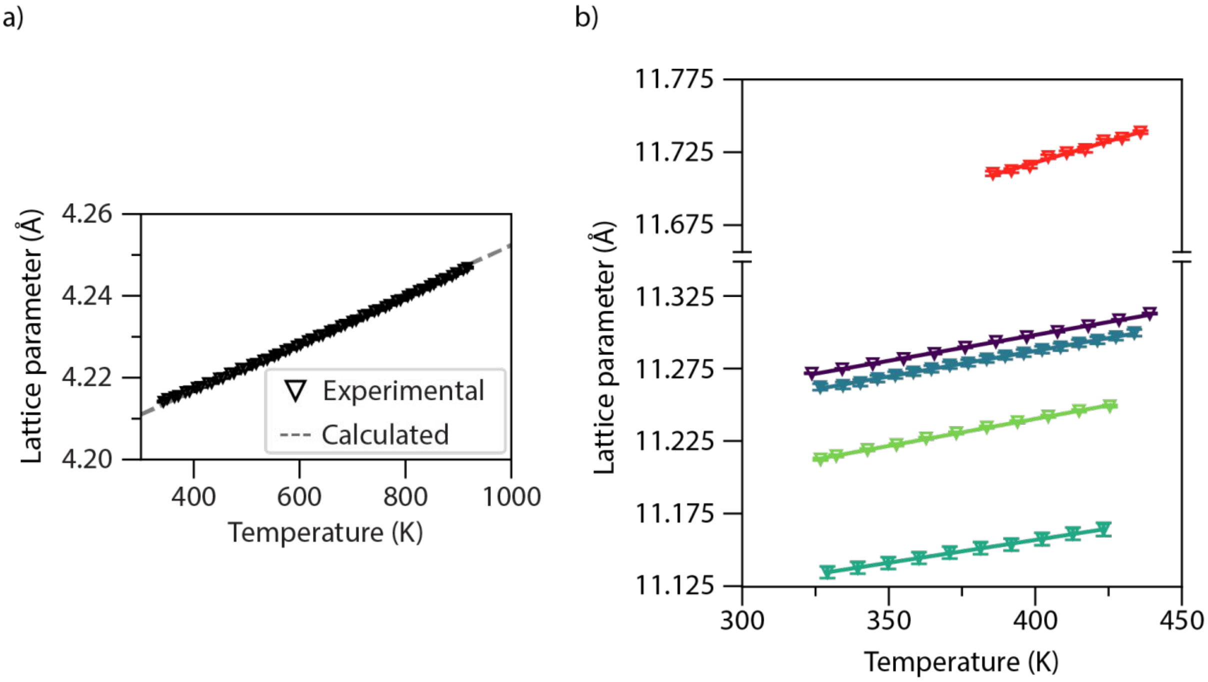

Figure S20. a) Temperature-dependent lattice parameter of MgO obtained using Rietveld refinement method of *in-situ* XRD patterns collected from 300 K to 900 K using a lab-based X-ray source. The dotted grey line represents eq. (1). **b)** Temperature-dependent lattice parameters of cubic alloyed elpasolites obtained using Rietveld refinement method of *in-situ* XRD patterns collected between 325 and 450 K using a lab-based X-ray source. From top to bottom the lines represent: $Cs_2AgBi(Br_{0.3}I_{0.7})_6$, $Cs_2AgBiBr_6$, $Cs_2Ag(Bi_{0.9}Fe_{0.1})Br_6$, $Cs_2Ag(Bi_{0.5}Sb_{0.5})Br_6$, and Cs2A$(Bi_{0.5}In_{0.5})Br_6$.

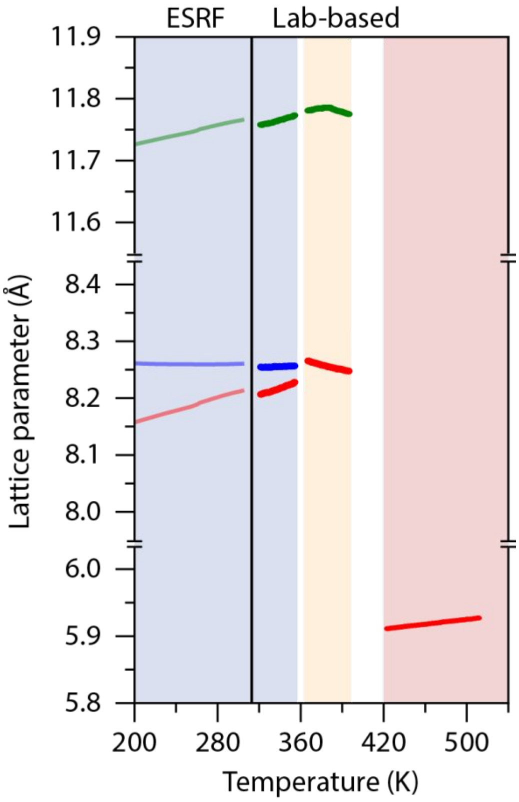

Figure S21. Lattice expansion of $CsPbBr_3$ over a temperature range of 200 to 540 K determined with synchrotron (<315 K) and lab-based X-ray sources (>315 K). For cubic CsPbBr3 (>420 K) the α was determined see **Table 1** in the main text.

References

(1) Dyadkin, V.; Pattison, P.; Dmitriev, V.; Chernyshov, D. A New Multipurpose Diffractometer PILATUS@SNBL. *J. Synchrotron Rad.* **2016**, *23*, 825–829.

(2) C. J. McMonagle; Allan, D. R.; Warren, M. R.; Kamenev, K. V.; Turner, G. F.; Moggach, S. A. High-Pressure Sapphire Capillary Cell for Synchrotron Single-Crystal X-Ray Diffraction Measurements to 1500 Bar. *J. Appl. Cryst.* **2020**, *53*, 1519–1523.

(3) J. Cosier; Glazer, A. M. A Nitrogen-Gas-Stream Cryostat for General X-Ray Diffraction Studies. *J. Appl. Crystallogr.* **1986**, *19*, 105–107.

(4) Marshall, K.; Emerich, H.; McMonagle, C.; Fuller, C.; Dyadkin, V.; Chernyshov, D.; van Beek, W. A New High Temperature, High Heating Rate, Low Axial Gradient Capillary Heater. *J. Synchrotron Radiat.* **2023**, *30*, 267–272.

(5) Swanson, D. K.; Prewitt, C. T. A New Radiative Single-Crystal Diffractometer Microfurnace Incorporating MgO as a High-Temperature Cement and Internal Temperature Calibrant. *J. Appl. Cryst.* **1986**, *19*, 1–6.
