# Supplementary material for: Which Ion Dominates the Temperature and Pressure Response of Halide Perovskites and Elpasolites?
Source: J Phys Chem Lett. 2023 Oct 2;14(40):9042–51. doi: 10.1021/acs.jpclett.3c02403 (PMC10577787; doi:10.1021/acs.jpclett.3c02403)
Supplement: Supplementary file 2 — jz3c02403_si_002.pdf [file jz3c02403_si_002.pdf]

jz-2023-02403h.R1

Name: Peer Review Information for "Which Ion Dominates Temperature and Pressure Response of Halide Perovskites and Elpasolites?"

First Round of Reviewer Comments

Reviewer: 1

Comments to the Author

The authors report on temperature and pressure-dependent X-ray diffraction analysis under relatively low pressures (up to 0.06 GPa) to assess the elastic properties of halide perovskites and double perovskites (elpasolites) of different representative compositions. They identify common trends in bulk moduli and thermal expansivity, with higher compressibility with the increase of halide ionic radii. They further show a minor effect of the A cations on elastic properties and identify phase transition effects in selected representatives. The mechanical properties of this class of materials are not very well studied and understood, which is of interest to their characteristics and stabilities in functional devices. This reviewer recommends considering it for publication upon addressing the following minor remarks.

- The authors use mechanochemical synthesis of the materials, which is well established for halide perovskites over the past years. However, this is not entirely clear for elpasolites (double perovskites), and the authors should refer to the previous reports relying on mechanochemical synthesis of these materials for clarity.

- In their pressure cell, the authors use a fluorinated inert liquid as a pressure-transmitting medium. They should comment on whether the liquid could affect material characteristics. In addition, they are using a certain pressure range without any justification, which would be of interest to comment on for a better understanding.

- The language is clear and coherent. The authors should, however, revise once more for minor formatting issues, such as the appropriate notation of space groups (e.g., use of italic letters etc.).

- The literature is comprehensive. It would be of interest to, however, mention more explicitly previous work on the mechanosynthesis and mechanical characteristics of double perovskites in the introduction for clarity.

Thank you for your consideration.

Reviewer: 2

Comments to the Author

The authors explore how lattice constants across various compositions change with temperature and applied pressure, using this information to derive the macroscopic mechanical properties of the materials under investigation. The manuscript is well-written, and its main message is clearly conveyed to the reader. Although I don't agree that all the conclusions of this work are novel, I recognize that a unique approach has been employed. Therefore believe that this work offers a fresh perspective and would be of benefit to the community if published. I recommend the manuscript for publication, pending minor revisions.

1. The powders used in this study were synthesized via ball milling, which differs significantly from the solution processing typically employed for thin-film deposition. Could the authors discuss how the conclusions relating to real-world devices may be drawn from materials synthesized through an unconventional method? How do the properties of ballmilled powders compare to those obtained through wet chemical methods?
2. I have some reservations about the novelty of this work. Previous studies have explored the evolution of mechanical properties in relation to temperature and composition (Phys. Rev. Lett., 121, 085502, J. Mater. Chem. C, 2018, 6, 7657 et al.). Furthermore, correlations between softening and the ionic radius of the halide atom (ACS Energy Lett., 2017, 2, 7, 1662), as well as softening near phase transitions (Phys. Rev. B, 105, 024306), have been reported. While the work does introduce novelty in the context of Elpasolites, could the authors clarify how their study distinguishes itself from existing literature?
3. The external compression shifts the peaks to higher 2theta values as discussed around Fig1, which is to be expected if the lattice constant decreases as a function of compressive pressure. However, that is not the case in Fig1a,b. 0.06 GPa purple curve shows peaks at lower 2theta angles compared to 0.004GPa (green) curve. Is there a mistake in the labelling of the figure?
4. The results presented in Figure 4 are interesting and offer valuable insights into the nature of the phase transitions for the compositions under study. To enhance clarity for the reader, it would be beneficial to specify the crystallographic meanings of the a, b, and c axes. It would be particularly helpful if the lattice constants could be converted into pseudocubic notation, allowing for the use of Glazer notation. This would clarify the orientation of, e.g. c-axis relative to any octahedral tilts. Additionally, it would be informative to visualize what the negative expansion of the c-axis, e.g. in $MAPbBr_3$, actually represents Does this imply that the lattice in the direction of the octahedral tilt (presuming the octahedra are tilted along the c direction in the tetragonal I4/mcm structure with an $a^0a^0c^-$ Glazer notation) is experiencing negative expansion? Elucidating these points would be advantageous for the reader's understanding.

Author's Response to Peer Review Comments:

Dear Dr.

Thank you for giving us the opportunity to revise our manuscript.
In this rebuttal, we outline our response to the reviewers' comments and how we have addressed these in the revised manuscript. The reviewers' comments are reproduced in blue, followed by our response in black, and changes to the manuscript are marked in yellow. In addition, we have addressed the editorial comments. We are convinced the revised manuscript is an improved version of the one we submitted previously, and we thank you for considering it for publication.

On behalf of all authors,

Dr. Muscarella and Dr. Hutter

Editorial requests

1. Please include annotated version(s) of your revised publication file(s) with colored text or highlights indicating the revisions that you have made, and upload them as “Supporting Information for Review Only." Please also upload “clean” copies for publication. (No highlighting, annotations, or colored text permitted.)

We have uploaded the revised version with highlights as "Supporting Information for Review Only" and a clean copy for publication.

2. Abstract: Shorten the abstract to 150 words or fewer.We have shortened the abstract to 149 words.

3. Headers: Remove the section heading(s) throughout the body of the manuscript (you can leave Methods, Abstract, and TOC Graphic headings).

We have removed the headings: Introduction, Result and Discussion and Conclusion.

4. TOC Graphic: Please resize the TOC graphic per journal guidelines (2 in x 2 in) and move to the correct position (on the same page as the abstract).

We have resized the TOC graphic and moved it on the same page as the abstract.

5. References: In both the main file and the supporting information, fix the style of all references to use JPCL formatting (check all references carefully). ***JPC Letters reference formatting requires that journal references should contain: () around numbers; author names; article title (titles entirely in title case or entirely in lower case); abbreviated journal title (italicized); year (bolded); volume (italicized); and pages (first-last). Book references should contain author names; book title (in the same pattern); publisher; city; and year. Websites must include date of access.

We have fixed the style of the references to use JPCL formatting.

6. References: URLs are not preferred references because website content can be modified and, consequently, the reference information may lack permanence.

We have removed reference 46, and renumbered the other references accordingly.

7. Supporting Information: Please number SI pages in the following format: “S1, S2…”We have numbered the SI pages in the correct format.

8. Graphics: One or more of your figures and tables includes a reference citation. Please confirm that this pertains only to data and not the figure itself. If it pertains to the use of a published image, permissions must be secured for any graphics NOT originally published by ACS or for Open Access content which permits reuse with credit only. Permission is needed if you are using another publisher's or copyright owner's figures/tables verbatim, adapting/modifying them, or using them in part. If the images are from an Open Access publisher that does not require permission for reuse, please confirm.

We confirm that the references included in the figures/table pertain only to data and not the figure itself.

Reviewer: 1

The authors report on temperature and pressure-dependent X-ray diffraction analysis under relatively low pressures (up to 0.06 GPa) to assess the elastic properties of halide perovskites and double perovskites (elpasolites) of different representative compositions. They identify common trends in bulk moduli and thermal expansivity, with higher compressibility with the increase of halide ionic radii. They further show a minor effect of the A cations on elastic properties and identify phase transition effects in selected representatives. The mechanical properties of this class of materials are not very well studied and

understood, which is of interest to their characteristics and stabilities in functional devices. This reviewer recommends considering it for publication upon addressing the following minor remarks.

We thank the reviewer for recognizing the importance of understanding the mechanical properties of these materials and the contribution of our work.

1. The authors use mechanochemical synthesis of the materials, which is well established for halide perovskites over the past years. However, this is not entirely clear for elpasolites (double perovskites), and the authors should refer to the previous reports relying on mechanochemical synthesis of these materials for clarity.

We thank the reviewer for the comment. We now include several references of halide perovskites and elpasolites synthesized using mechanochemical synthesis such as:

(33) Rodkey, N.; Kaal, S.; Sebastia-Luna, P.; Birkhölzer, Y. A.; Ledinsky, M.; Palazon, F.; Bolink, H. J.; Morales-Masis, M. Pulsed Laser Deposition of Cs2AgBiBr6: From Mechanochemically Synthesized Powders to Dry, Single-Step Deposition. *Chem. Mater.* **2021**, *33* (18), 7417–7422. https://doi.org/10.1021/acs.chemmater.1c02054.

(35)

(34) Palazon, F.; El Ajjouri, Y.; Bolink, H. J. Making by Grinding: Mechanochemistry Boosts the Development of Halide Perovskites and Other Multinary Metal Halides. *Adv. Energy Mater.* **2020**, *10*, 1902499. https://doi.org/10.1002/aenm.201902499.

Breternitz, J.; Levcenko, S.; Hempel, H.; Gurieva, G.; Franz, A.; Hoser, A.; Schorr, S. Mechanochemical Synthesis of the Lead-Free Double Perovskite Cs2[AgIn]Br6 and Its Optical Properties. *JPhys Energy* **2019**, *1* (2). https://doi.org/10.1088/2515-7655/ab155b.

(36) Karmakar, A.; Askar, A. M.; Bernard, G. M.; Terskikh, V. V; Ha, M.; Patel, S.; Shankar, K.; Michaelis, V. K. Mechanochemical Synthesis of Methylammonium Lead Mixed − Halide Perovskites: Unraveling the Solid-Solution Behavior Using Solid-State NMR. *Chem. Mater.* **2018**, *30*, 2309–2321. https://doi.org/10.1021/acs.chemmater.7b05209.

We have included these references on page 7:

Microcrystalline powder samples of $MAPb(Cl_{1-x}Br_x)_3$, $MAPb(I_{1-x}Br_x)_3$, $CsPbCl_3$, $-Br_3$, and $-I_3$, and several elpasolites (based on Cl^-, Br^-, Bi^{3+} and/or Fe^{3+}, Sb^{3+}, In^{3+}) were made via synthesis in [33-36] mechanochemical a ball mill,[10,] as described in the **Experimental Section** in the **Supporting Information**.

In addition, we have again referred to reference 10 here, where the same mechanochemical synthesis approach was used to obtain mixed-metal elpasolites.

2. In their pressure cell, the authors use a fluorinated inert liquid as a pressure-transmitting medium. They should comment on whether the liquid could affect material characteristics. In addition, they are using a

certain pressure range without any justification, which would be of interest to comment on for a better understanding.

We thank the reviewer for the comment. The liquid was chosen because it is thermally and chemically stable and compatible with all the perovskites studied in the work. This liquid (and similar class of liquids, e.g., FC-72) has also been used in the community of perovskites for many types of experiments due to its characteristics (e.g., https://doi.org/10.1038/s41560-022-01154-y , https://doi.org/10.1088/2515-7639/ab9aac , https://doi.org/10.1021/acsenergylett.0c01474)

We have now included the following information on page S2 of the Supporting Information:

The FC-770 liquid is thermally and chemically stable and compatible with all the perovskites studied in this work. Furthermore, it evaporates without leaving residuals on the sample.

As for the pressures used, we have decided to perform the experiments at very mild pressure to emulate the strain that can be potentially induced during the manufacturing process, as already mentioned on page 5 of the main text:

The deformations induced in halide perovskite films during the solution-processed deposition are comparable to the exertion of mild pressure (<0.5 GPa).[32]

As depicted in Figure 1d from reference 32, shown below, the typical annealing temperatures used in the deposition of thin films of $MAPbI_3$ (around 100 °C) result in a film stress below 60 MPa (equivalent to 0.06 GPa). Therefore, our work is focused on examining the pressure range from ambient conditions to 0.060 GPa, as it holds greater significance for thin films production.

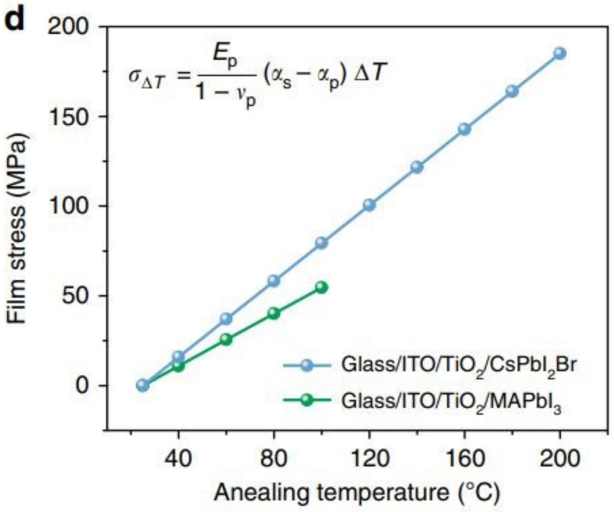

We clarify this in the main text on page 5-6:

Given that the typical annealing temperatures applied during the deposition of halide perovskite thin films (100°C) yield film stress levels below 0.06 GPa,[32] our work, conducted through synchrotronbased powder X-ray diffraction (XRD), explored the structural properties of halide perovskites and elpasolites at elevated pressures up to 0.060 GPa, using a hydrostatic pressure cell.

3. The language is clear and coherent. The authors should, however, revise once more for minor formatting issues, such as the appropriate notation of space groups (e.g., use of italic letters etc.). We have revised again the manuscript for fixing the minor formatting issues.

4. The literature is comprehensive. It would be of interest to, however, mention more explicitly previous work on the mechanosynthesis and mechanical characteristics of double perovskites in the introduction for clarity.

See our response to comment 2 from reviewer 1, and comment 1 from reviewer 2.

Reviewer 2

The authors explore how lattice constants across various compositions change with temperature and applied pressure, using this information to derive the macroscopic mechanical properties of the materials under investigation. The manuscript is well-written, and its main message is clearly conveyed to the reader. Although I don't agree that all the conclusions of this work are novel, I recognize that a unique approach has been employed. Therefore believe that this work offers a fresh perspective and would be of benefit to the community if published. I recommend the manuscript for publication, pending minor revisions.

We thank the reviewer for recognizing the unique approach we have used in this work.

1. The powders used in this study were synthesized via ball milling, which differs significantly from the solution processing typically employed for thin-film deposition. Could the authors discuss how the conclusions relating to real-world devices may be drawn from materials synthesized through an unconventional method? How do the properties of ball- milled powders compare to those obtained through wet chemical methods?

Both ball-milling and solution-processing have proven successful in obtaining materials with the desired perovskite or elpasolite crystal structure. The main difference between these synthesis procedures is the crystallinity, which is typically higher for solution-processing because this typically involves controlled nucleation and growth processes (e.g., using antisolvents). The resulting crystals tend to have fewer defects, while the ball milling method could introduce defects and disorder due to the mechanical forces involved. For an elaborate description of mechanochemical synthesis, we refer to ref 36 in the revised manuscript.

36. Palazon, F.; El Ajjouri, Y.; Bolink, H. J. Making by Grinding: Mechanochemistry Boosts the Development of Halide Perovskites and Other Multinary Metal Halides. *Adv. Energy Mater.* **2020**, *10*, 1902499. https://doi.org/10.1002/aenm.201902499.

As mentioned in the manuscript, thin films may suffer from strain introduced by the substrate during growth. Hence, we decided here to use powder materials to study the intrinsic thermoelastic properties of the materials of interest. However, it is indeed possible that the differences in strain between ballmilled powders and solution-processed films lead to subtle changes in these properties. As an example, comparing our results to previous reports on elpasolites fabricated with wet chemical methods, we did not detect the low T phase transition for $Cs_2AgBiBr_6$. Nevertheless, it is worth noting that other compositions studied in this study, such as $CsPbCl_3$ and $MAPbI_3$, undergo phase transitions at temperatures comparable to those observed in thin films. Specifically, $CsPbCl_3$ undergoes a phase transition between 310-320 K in thin film form, while we observe a phase transition at 325 K in our powder samples. Similarly, in the case of $MAPbI_3$, a phase transition occurs between 315-330 K in thin films, whereas we detect a phase transition at 330 K

in our powder samples. The same trend applies to $CsPbBr_3$, where the expected and observed phase transition occurs at 360 K, and $MAPbCl_3$, where the expected and observed phase transition takes place at 160 K.

We have made an explicit statement about this in the revised manuscript on page 13:

(57)

Note that previous studies on $Cs_2AgBiBr_6$ thin films have reported a cubic-to-tetragonal phase transition at 122 K.[57] The absence of this phase transition (at least down to 100 K) for the mechanochemically synthesized powders studied here may be related to differences in strain between the powders and thin films. Note that $Cs_2AgBiBr_6$ stands out as the sole exception; the other compositions exhibit the expected phase transitions at their respective temperatures.

Schade, L.; Wright, A. D.; Johnson, R. D.; Dollmann, M.; Wenger, B.; Nayak, P. K.; Prabhakaran, D.; Herz, L. M.; Nicholas, R.; Snaith, H. J.; Radaelli, P. G. Structural and Optical Properties of Cs 2 AgBiBr 6 Double Perovskite. *ACS Energy Lett.* **2019**, *4* (1), 299–305. https://doi.org/10.1021/acsenergylett.8b02090.

In addition, we have specified the reason of chosing to study the powder instead of the thin films, on page 4:

Powders are employed as a model system because they do not experience strain from the substrate.

2. I have some reservations about the novelty of this work. Previous studies have explored the evolution of mechanical properties in relation to temperature and composition (Phys. Rev. Lett., 121, 085502, J. Mater. Chem. C, 2018, 6, 7657 et al.). Furthermore, correlations between softening and the ionic radius of the halide atom (ACS Energy Lett., 2017, 2, 7, 1662), as well as softening near phase transitions (Phys. Rev. B, 105, 024306), have been reported. While the work does introduce novelty in the context of Elpasolites, could the authors clarify how their study distinguishes itself from existing literature?

   Unlike the existing literature, which typically focuses on either temperature-dependent (Tdependent) or pressure-dependent (P-dependent) studies, our research is the first report of a comprehensive investigation that considers both pressure and temperature variations simultaneously *under mild pressure conditions*. This novel approach allows us to gain a deeper understanding of the elastic response of these materials during thin film fabrication, where both strain and temperature are involved. Furthermore, our study explores a broad range of materials using a consistent synthesis and analysis methodology across multiple compositions, providing a fair comparison for an extensive set of materials: much larger than is commonly found in literature. By fabricating and measuring the compositions with the same approach, we mitigate the challenges posed by variations in synthesis methods and techniques used in different laboratories, enabling us to draw more robust and general conclusions about the fundamental properties and behaviors of these materials.

In response to the previous and present comment from reviewer 2, we have added the following sentence in the introduction on page 4:

These materials were synthesized as powders using mechanochemical synthesis in a ball, which has been successfully used to make both perovskite and elpasolite materials.[10,33–36] By using the same fabrication and measurement approach for a large number of compositions, we exclude effects from sample type or varying synthesis procedure used in different laboratories, enabling us to draw robust and general conclusions about the fundamental properties and behaviors of these materials.

3. The external compression shifts the peaks to higher 2theta values as discussed around Fig1, which is to be expected if the lattice constant decreases as a function of compressive pressure. However, that is not the case in Fig1a,b. 0.06 GPa purple curve shows peaks at lower 2theta angles compared to 0.004GPa (green) curve. Is there a mistake in the labelling of the figure?

We appreciate the reviewer for bringing the error in Figure 1 to our attention. The labels are indeed swapped. We have now fixed the issue and replaced the figure accordingly.

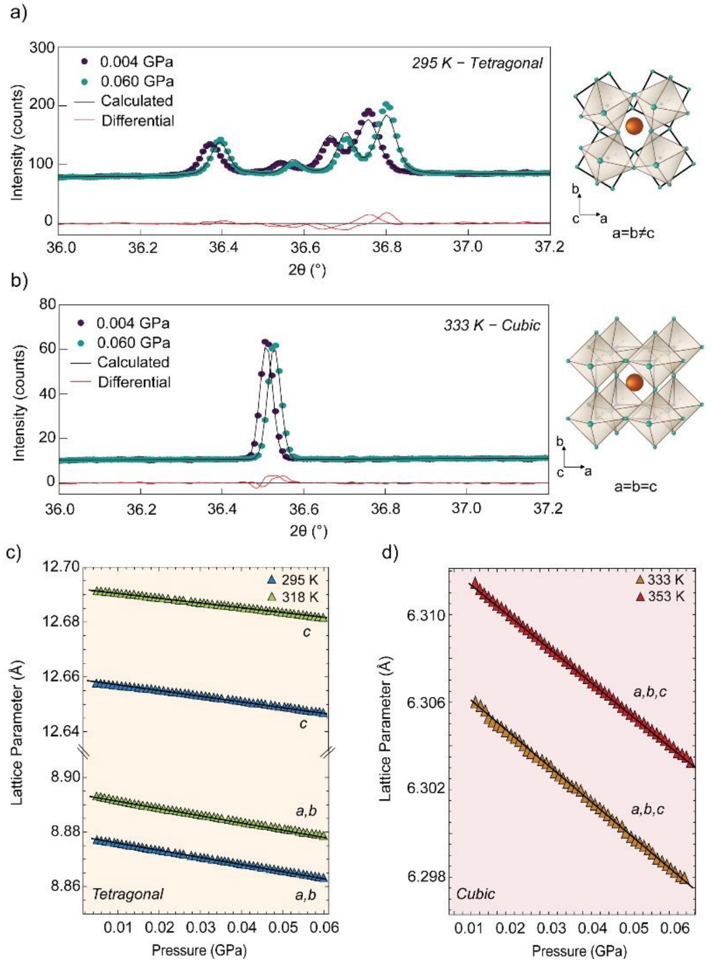

4. The results presented in Figure 4 are interesting and offer valuable insights into the nature of the phase transitions for the compositions under study. To enhance clarity for the reader, it would be beneficial to specify the crystallographic meanings of the a, b, and c axes. It would be particularly helpful if the lattice constants could be converted into pseudocubic notation, allowing for the use of Glazer notation. This would clarify the orientation of, e.g. c-axis relative to any octahedral tilts.

Additionally, it would be informative to visualize what the negative expansion of the c-axis, e.g. in MAPbBr3, actually represents Does this imply that the lattice in the direction of the octahedral tilt (presuming the octahedra are tilted along the c direction in the tetragonal I4/mcm structure with an a0a0c- Glazer notation) is experiencing negative expansion? Elucidating these points would be advantageous for the reader's understanding.

We thank the reviewer for these suggestions. We agree that the pseudocubic notation provides a more gradual change of the lattice parameter despite a phase transition. Therefore, we included it in an additional figure in the SI (**Figure S14)**. We, however, prefer the original representation in the main manuscripts as the phase transitions are highlighted.

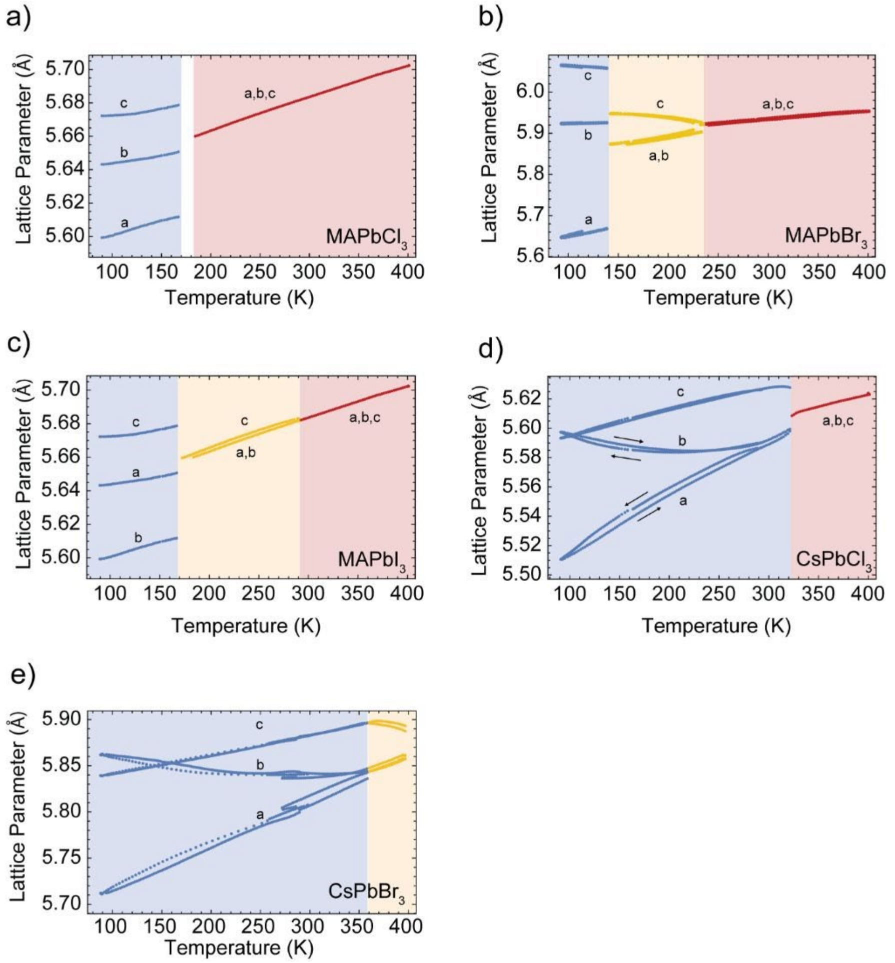

Figure S14. Pseudocubic lattice parameters of **a)** $MAPbCl_3$, **b)** $MAPbBr_3$, **c)** $MAPbI_3$, **d)** $CsPbCl_3$, **e)** $CsPbBr_3$ between 90 K and 400 K. The blue, yellow and red regions correspond to the orthorhombic, tetragonal and cubic phase, respectively. The regions in white correspond to the temperature range where two phases coexist in the XRD patterns. The black arrows indicate the direction of temperature variation, *i.e.* cooling (arrow down) and heating (arrow up). Solid lines represent the fit for the linear thermal expansivity.

The use of the Glazer notation will indeed provide interesting insights in the tilting, or folding, of the octahedra in the non-cubic phases as a function of pressure and temperature. This, however, requires resolving the (changing) bond angles from the X-ray diffraction data. As we describe in the experimental section, we did not take the individual atomic coordinates, nor the interatomic distances, into account in our refinement method. Our temperature-dependent measurements are likely of sufficient quality to resolving the bond angles, however doing this for all composition would require extensive further data analysis. Such analysis would definitely be interesting for a follow-up study, which is why we published the dataset open access, see statement in the manuscript.

Data and fit procedures reported in this study can be accessed at https://data.esrf.fr/doi/10.15151/ESRF-ES-1022932247 (raw data) and 10.24416/UU01-W60H58 (processed data) and are available under a CC-BY Creative Commons Attribution 4.0 International license.
